# The Role of G Protein-Coupled Receptors in the Right Ventricle in Pulmonary Hypertension

**DOI:** 10.3389/fcvm.2018.00179

**Published:** 2018-12-17

**Authors:** Gayathri Viswanathan, Argen Mamazhakypov, Ralph T. Schermuly, Sudarshan Rajagopal

**Affiliations:** ^1^Division of Cardiology, Department of Medicine, Duke University Medical Center, Durham, NC, United States; ^2^Department of Internal Medicine, Member of the German Center for Lung Research (DZL), Justus Liebig University of Giessen, Giessen, Germany

**Keywords:** right ventricle, G protein-coupled receptor, pulmonary hypertension, remodeling, dysfunction

## Abstract

Pressure overload of the right ventricle (RV) in pulmonary arterial hypertension (PAH) leads to RV remodeling and failure, an important determinant of outcome in patients with PAH. Several G protein-coupled receptors (GPCRs) are differentially regulated in the RV myocardium, contributing to the pathogenesis of RV adverse remodeling and dysfunction. Many pharmacological agents that target GPCRs have been demonstrated to result in beneficial effects on left ventricular (LV) failure, such as beta-adrenergic receptor and angiotensin receptor antagonists. However, the role of such drugs on RV remodeling and performance is not known at this time. Moreover, many of these same receptors are also expressed in the pulmonary vasculature, which could result in complex effects in PAH. This manuscript reviews the role of GPCRs in the RV remodeling and dysfunction and discusses activating and blocking GPCR signaling to potentially attenuate remodeling while promoting improvements of RV function in PAH.

## Introduction

Right ventricular (RV) dysfunction and failure predict mortality in a number of cardiopulmonary diseases including pulmonary arterial hypertension (PAH) (1), heart failure (2–4) and chronic obstructive pulmonary disease (COPD) (5, 6). Initially, the RV undergoes favorable (adaptive) remodeling characterized by an increase in RV wall thickness, dilation and mass mediated by cardiomyocyte hypertrophy and moderate extracellular matrix deposition to maintain its contractility to the increased afterload (7, 8). At some point in the course of the disease, the compensatory mechanisms of the RV are exhausted and the RV undergoes maladaptive remodeling with RV dilation and dysfunction (9), which is characterized by insufficient angiogenesis (10), excessive inflammation (11), and fibrosis (12). Despite similar pulmonary hemodynamics across different causes of PAH, such as that due to congenital heart disease or scleroderma, there are wide variations in outcomes in PAH depending on the etiology (13). For example, survival and functional status of PAH patients due to congenital heart diseases (CHD) are better than those of IPAH patients (14, 15), which may be explained by the compensated RV function and favorable (adaptive) RV remodeling (increased wall thickness) due to longstanding PAH (16). For these reasons, many groups have postulated that the RV, and not the pulmonary circulation, should be the major target for treatment in PAH (17).

In left heart failure, after treatment of excess afterload (with systemic blood pressure targets of <130/80), treatments target the left ventricle itself. Many evidence-based therapies for left heart failure specifically target G protein-coupled receptors (GPCRs) expressed on cardiomyocytes (18). GPCRs represent the largest family of membrane receptors involved in signal transduction and ~34% of FDA-approved drugs block or activate different GPCRs (19). Altered GPCR signaling pathways play crucial roles in the pathogenesis of major cardiovascular diseases such as systemic hypertension, coronary artery diseases (CAD), and left heart failure, and agents targeting these GPCRs serve as cornerstone treatment strategies in these diseases (20). Similarly, several GPCR signaling pathways are dysregulated in PAH and serve as targets for drugs, such as endothelin receptor antagonists (ERAs) and prostanoids (21). Many treatments for PAH have little effect on pulmonary artery pressure, as a meta-analysis reveals that in PAH patients, mPAP decreases only by 2.87 mmHg during 14.3 weeks of treatment (22). Apart from effecting pulmonary vasculature, some of these same treatments have been shown to have direct effect on the RV in preclinical studies. However, a meta-analysis of clinical trials showed that 12 week treatment with current PAH therapies do not have a favorable direct effect on right heart function (23). Moreover, some of these treatment strategies for PAH may even be detrimental to the right ventricle. For example, bosentan exerts negative inotropic effect on the hypertrophied RV in isolated perfused rat heart (24). Similarly, several synthetic prostanoids impair RV function in the hypertrophied heart while improving RV function in the healthy rat heart (25). Similarly, development of peripheral edema in patients taking PAH specific therapy may indicate the deterioration of RV failure due to the treatment (24, 26). Notably, right heart function is a critical prognostic determinant in patients with PAH, as patients with impaired RV performance despite a significant pulmonary vasodilatory effect of the therapy (27). Thus, developing therapies focusing on RV function in PAH may improve symptoms, quality of life, hemodynamics, and survival. In this review, we highlight GPCR drug targets in the RV, the effects of targeting them in preclinical and clinical studies, and the challenges around developing these therapeutics.

## GPCR Signaling

GPCRs are the most common receptors encoded in the genome, comprising >1% of the coding human genome with ~800 members and expressed within every organ system (28). GPCRs share a common architecture with an extracellular N-terminal sequence, seven transmembrane-spanning domains, and an intracellular C-terminal domain. GPCRs sense a wide range of extracellular stimuli, including proteins, small molecules, hormones, neurotransmitters, ions, and light. GPCR signaling is primarily controlled by three protein families: G proteins, G protein receptor kinases (GRKs), and β-arrestins. These proteins perform distinct functions at the receptor (29). Upon stimulation with an agonist, GPCRs activate heterotrimeric G proteins by catalyzing the exchange of GTP for GDP on Gα subunits of the heterotrimeric G-protein. This leads to dissociation of the heterotrimeric complex into Gα and Gβγ subunits. The dissociated subunits have different roles, with the Gα subunit regulating second messenger effectors such as cyclic adenosine monophosphate (cAMP—promoted by Gs and inhibited by Gi/o), inositol triphosphate (IP3—promoted by Gq/11), diacylglycerol (DAG—promoted by Gq/11), while the Gβγ subunit can modulate other receptors and channels, such as inward rectifying potassium channels. After ligand binding, the receptor is phosphorylated by a number of kinases, primarily by GRKs, on its C-terminus and cytoplasmic loops (30), which enhance β-arrestin binding to the receptor. β-arrestins mediate receptor desensitization (31), the process whereby repeated stimulation decreases the signaling response over seconds to minutes, and receptor internalization (32–34). This results in receptor downregulation, a decrease in receptor membrane expression over minutes to hours with trafficking of the receptors to proteasomes or lysosomes. In addition to acting as negative regulators of G protein signaling, β-arrestins also couple to numerous signaling mediators including kinases and transcription factors by acting as adaptors and scaffolds (35–41). These pathways are separate from classical G protein signaling, but can involve similar signaling cascades that are often temporally distinct.

## The Right Ventricle Is Not Just A Wimpy Left Ventricle

The right ventricle is different from the left ventricle from the point of embryology (42), structure (43, 44), functionality (45) as well as sarcomere structure (46). The normal pulmonary circulation represents a low-resistance, high compliance load to the right ventricle (RV) and a low pressure is sufficient to pump blood to the lungs for oxygenation. RV function is reflected in its structure. The RV is thin-walled and crescent-shaped compared to the left ventricle (LV), which has a circular/ellipsoidal cross-section that combined with larger muscle mass can generate higher pressures (17). Similarly, at a molecular level, there are significant differences between the RV and LV in the expression of genes known to be involved in the response to pressure overload and failure (7), with different RV and LV responses to certain effectors. For example, α_1_-adrenergic receptor (α_1_AR) agonists increase LV contractility, but may decrease RV contractility (47). Chronic infusion of norepinephrine induces hypertrophy in LV but not in RV (48). miRNA profiling in hypertrophy to failure revealed several notable differences between RV and LV miRNAs. These include miRNAs that are linked to cell proliferation, metabolism, survival, extracellular matrix turnover, and impaired proteasomal function. For example, miRNA 93, miRNA 148a, and miRNA 28 were upregulated in RV hypertrophy/failure and downregulated or unchanged in LV hypertrophy/failure (49). Therefore, the findings from left heart physiology and pathophysiology cannot be simple extrapolated to the right heart (50, 51).

## Role of GPCRs in RV Remodeling And Dysfunction

Many GPCRs have been studied in animal studies of RV hypertrophy and failure. We have summarized a non-comprehensive list of preclinical studies of RV hypertrophy and failure that quantified changes in GPCR expression in the RV (Table 1), changes in the expression of GPCR ligands in the RV (Table 2), and the effects of treatment with GPCR ligands (Table 3). Below we focus on a number of receptors that have been studied for their effects on RV function.

**Table 1:** Summary of studies evaluating GPCR expression in the RV in PAH patients or in different models of right heart hypertrophy/failure.

**Disease model/disease**	**Species/subjects**	**Methods**	**GPCRs**	**References**
PAH patients	Human	LBA	↑ endothelin-1 receptor type A (ET_R_A)	(52)
PAH patients	Human	LBA	↓ endothelin-1 receptor type B (ET_R_B)	(52)
PAH patients	human	IFS	↑ endothelin-1 receptor type A (ET_R_A)	(24)
PAH patients	Human	WB	↑α-7nAchR	(53)
PAH patients	Human	WB	↔m2AchR	(53)
MCT induced RV remodeling	Rats	LBA	↓β-adrenergic receptors (β-ARs)	(54)
MCT induced RV remodeling	Rats	WB	↓β-adrenergic receptors (β1-AR, β2-AR)	(55)
MCT induced RV remodeling	Rats	LBA	↓β-adrenergic receptors (β1-AR, β2-AR)	(56)
MCT induced RV remodeling	Rats	LBA	↓β-adrenergic receptors (β-ARs)	(57)
HOX induced RV remodeling	Rats	PCR	↓β1-adrenergic receptor (β1-AR)	(58)
HOX induced RV remodeling	Rats	PCR	↔β2-adrenergic receptor (β2-AR)	(58)
HOX induced RV remodeling	Rats	LBA	↓β-adrenergic receptors (β-ARs)	(58)
MCT induced RV remodeling	Dogs	LBA	↑β-adrenergic receptors (β-ARs)	(59)
MCT induced RV remodeling	Dogs	LBA	↑α1-adrenergic receptor (α1-AR)	(59)
MCT induced RV remodeling	Rats	LBA	↓α1-adrenergic receptor (α1-AR)	(56)
MCT induced RV remodeling	Rats	IFS	↑ endothelin-1 receptor type A (ET_R_A)	(24)
HOX induced RV remodeling	Rats	GeneChip analysis, PCR, WB	↑ endothelin-1 receptor type B (ET_R_B)	(60)
MCT induced RV remodeling	Rats	LBA	↓ endothelin-1 receptor type A (ET_R_A)	(52)
MCT induced RV remodeling	Rats	LBA	↑ endothelin-1 receptor type B (ET_R_B)	(52)
MCT induced RV remodeling	Rats	PCR	↑ endothelin-1 receptor type A and B (ET_R_B)	(61)
MCT induced RV remodeling	Rats	LBA	↑ endothelin-1 receptor type A and B (ET_R_B)	(62)
HOX induced RV remodeling	Rats	PCR	↑ endothelin-1 receptors (ET_R_A and ET_R_B)	(63)
PAB induced RV remodeling	Rabbits	IFS	↑ endothelin-1 receptor type A and B (ET_R_B)	(64)
HOX induced RV remodeling	Rats	PCR	↓ angiotensin-II receptor (AT_2_R)	(65)
MCT induced RV remodeling	Rats	PCR	↑ angiotensin-II receptor (AT_1_R) (at initial stages)	(65)
MCT induced RV remodeling	Rats	LBA	↑ angiotensin- II receptor (AT_2_R)	(65)
MCT induced RV remodeling	Rats	LBA	↑ angiotensin-II receptor (AT_1_R) (at initial stages)	(65)
MCT induced RV remodeling	Ovariectomized female rats	PCR	↑ angiotensin-II receptors (AT_1_R, AT_2_R)	(66)
HOX induced RV remodeling	Rats	WB	↑ angiotensin-II receptors (AT_1_R)	(67)
PAB induced RV remodeling	Rats	PCR	↓ angiotensin-II receptors (AT_1_R)	(68)
MCT induced RV remodeling	Rats	PCR	↔ angiotensin-II receptors (AT_1_R and AT_2_R)	(69)
MCT induced RV remodeling	Rats	LBA	↑ angiotensin-II receptor (AT_1_R)	(70)
SuHx induced RV remodeling	Rats	PCR	↓ APJ-receptor	(71)
MCT induced RV remodeling	Rats	PCR	↓ APJ-receptor	(72)
HOX induced RV remodeling	Rats	GeneChip analysis	↑ chemokine receptor (CXCR4)	(60)
PAB induced RV remodeling	Dogs	PCR	↑ chemokine receptor (CCR2)	(73)
PE model	Rats	PCR	↑ chemokine receptor (CCR1 and CXCR4)	(74)
MCT induced RV remodeling	Rats	LBA	↔ muscarinic receptors	(57)

*PAH, pulmoinary arterial hypertension; MCT, monocrotaline; HOX, hypoxia; SuHx, sugen plus hypoxia; LBA, ligand binding assay; PCR, polymerase chain reaction; WB, western blot; IFS, immunofluorescent staining*.

**Table 2:** Summary of studies evaluating the expressions of GPCR ligands and ligand modulators in the RV in different models of right heart hypertrophy/failure.

**Disease model**	**Species/subjects**	**Method**	**GPCR modulator/ligand**	**References**
MCT induced RV remodeling	Rats	HPLC	↑ epinephrine	(54)
MCT induced RV remodeling	Rats	HPLC	↑ norepinephrine	(54)
MCT induced RV remodeling	Rats	HPLC	↓ norepinephrine	(75)
MCT induced RV remodeling	Rats	Endothelin RIA assay	↓ endothelin-1	(76)
HOX induced RV remodeling	Rats	PCR	↔ endothelin-1	(63)
MCT induced RV remodeling	Rats	PCR	↑ endothelin-1	(61)
MCT induced RV remodeling	Rats	PCR	↑ endothelin-1	(77)
MCT induced RV remodeling	Rats	Endothelin RIA assay	↑ endothelin-1	(62)
MCT induced RV remodeling	Rats	PCR	↑ endothelin-1	(78)
MCT induced RV remodeling	Rats	IFS	↑ endothelin-1	(24)
MCT induced RV remodeling	Rats	ACE activity assay	↑ ACE activity	(79)
PAB induced RV remodeling	Dogs	ACE activity assay	↔ ACE activity	(80)
MCT induced RV remodeling	Rats	PCR	↑ renin	(69)
MCT induced RV remodeling	Rats	PCR	↑ angiotensinogen	(69)
HOX induced RV remodeling	Rats	Apelin content assay	↑ apelin	(81)
SuHx induced RV remodeling	Rats	PCR	↓ apelin	(82)
MCT induced RV remodeling	Rats	PCR	↓ apelin	(71)
MCT induced RV remodeling	Rats	PCR	↓ apelin	(72)
SuHx induced RV remodeling	Rats	PCR	↓ apelin	(83)
PAB induced RV remodeling	Mice	PCR	↑ CXCL10, CXCL6, CCL8, CX3CL1, CCL5, CXCL16, CCL2, CCL3	(73)
PE model	Rats	PCR	↑ CXCL1 and CXCL2	(84)
PE model	Rats	PCR	↑ CXCL1, CXCL2	(85)
PE model	Rats	PCR	↑ CC-chemokine genes (CCL2, 3, 4, 6, 7, 9, 17, 20, 27), CXC-chemokine genes (CXCL1, 2, 9, 10, 16)	(74)
PAB induced RV remodeling	Pigs	Microarray	↑ CCL2, CXCL6, CXCL2	(86)
PE model	Rats	PCR	↓ XCL-1 and CXCL-12	(74)

*PAH, pulmonary arterial hypertension; PAB, pulmonary artery banding; MCT, monocrotaline; SuHx, sugen plus hypoxia; HOX, hypoxia; PE, pulmonary embolism; PCR, polymerase chain reaction; IFS, immunofluorescent staining; HPLC, high performance liquid chromatography*.

**Table 3:** Summary of studies evaluating the effects of treatment with GPCR ligands in preclinical models of right heart hypertrophy/failure.

**Agent**	**Mechanism of action**	**Treatment option**	**Study design**	**Agent application details**	**Main effects of the drug on PA and the RV**	**References**
Captopril	ACE-1 inhibitor	Preventive	HOX rats (14 days)	Osmotic minipump, 20 mg/kg/day (days 0–14)	- Prevented the rise in PAP (↓ mPAP) - Prevented RV hypertrophy (↓RV/BW) - Prevented PA remodeling (↓muscularized PAs)	(87)
Captopril	ACE-1 inhibitor	Preventive	MCT rats (25 days)	Oral gavage, 30 mg/kg/day (days 1–25)	- Did not prevent the rise in PAP (↔PAP) - Preserved RV function (↑TAPSE) - Prevented the changes of modulators of RV ECM (↓MMP2 and MMP9 expressions, ↓MMP2 and MMP9 activities)	(88)
Enalapril	ACE-1 inhibitor	Preventive	MCT rats (28 days)	Oral gavage, 25 mg/kg/day (days 1–28)	- Did not prevent the rise in PAP (↔RVSP) - Prevented RV hypertrophy [↓RV/(LV+S)] - Prevented the rise in plasma markers of hypertrophy (↓ANP) - Prevented the change in RV norepinephrine content (↑NE) - Decreased mortality - Preserved RV enzymatic activity (↑CK activity, ↑LD-1 activity)	(75)
Enalapril	ACE-1 inhibitor	Preventive	MCT rats (5 weeks)	Drinking water, 4.4 mg/kg/day (5 weeks)	- Prevented RV hypertrophy [↓RV/(LV+S)]	(89)
Ramipril	ACE-1 inhibitor	Preventive	PAB rabbits (21 days)	Injection (i.p.), 37.5 mg/kg (1 hour after surgery), further in drinking water (1 mg/kg/day) (21 days)	- Did not prevent the rise in PAP (↔RVSP) - Did not prevent RV hypertrophy (↔RV/BW) - Preserved papillary cardiomyocyte contractility - Preserved RV enzymatic activity (↓Gαq, ↓Gαi1/2)	(70)
Losartan	AT_1_R blocker	Curative	PAB rats (7 weeks)	Oral gavage, 20 mg/kg/day (6 weeks)	- Did not have influence on any of the measured parameters of RV	(90)
Losartan	AT_1_R blocker	Preventive	PAB rabbits (21 days)	Injection (i.p.), 0.25 mg/kg, 1 h after surgery, then 50 mg/kg/d in the drinking water	- Did not prevent the rise in PAP (↔RVSP) - Did not prevent RV hypertrophy (↔RV/BW) - Preserved papillary cardiomyocyte contractility - Preserved RV enzymatic activity (↓Gαq, ↓Gαi1/2)	(70)
Losartan	AT_1_R blocker	Curative	MCT rats (25 days)	Vanilla pudding, daily, 20 mg/kg	- Reduced PAP (↓RVSP) - Reduced PVR (↓PVR) - Reduced RV dilation (↓RVEDD) - Did not decrease RVWT - Did not improve RV function (↔CO, ↔TAPSE) - Did not increase RV contractility (↔Ees) - Improved RV diastolic function (↓Eed) - Reduced RV afterload (↓Ea) - Reduced PA remodeling (↔ wall thickness) - Did not decrease RV cardiomyocyte hypertrophy (↔RV CSA)	(91)
Losartan	AT_1_R blocker	Preventive	HOX rats (14 days)	Osmotic minipump, 20 mg/kg/day (days 0–14)	- Prevented the rise in PAP (↓ mPAP) - Prevented RV hypertrophy (↓RV/BW) - Prevented PA remodeling (↓muscularized PAs)	(87)
Candesartan	AT_1_R blocker	Preventive	PAB dogs (60 days)	Oral, 1 mg/kg/day (60 days)	- Prevented thickening RV wall thickness - Decreased RV fibrosis - Decreased RV cardiomyocyte diameter - Increased circulating levels of RAAS members (↑renin, ↑angI, ↑AngII)	(80)
Telmisartan	AT_1_R blocker	Preventive	MCT rats (25 days)	Oral in distilled water, 3 mg/kg/day (24 days)	- Prevented RV hypertrophy (↓RV/Tibia) - Preserved RV function (↑TAPSE) - Prevented the changes of regulators in RV ECM remodeling (↓MMP2, ↓MMP9, TGF-β1)	(92)
Valsartan	AT_1_R blocker	Preventive	MCT rats (21 days)	Oral gavage, 20 mg/kg/day (21 days)	- Prevented the rise in PAP (↓RVSP) - Preserved RV function (↑RVEF, ↑TAPSE) - Prevented PA remodeling (↓wall thickness) - Prevented RV hypertrophy [↓RV/(LV+S)] - Did not prevent RV fibrosis (↔RV collagen area) - Prevented RV cardiomyocyte apoptosis (↓TUNEL positive cells, ↓Fas, ↓caspase-3, ↓bax, ↑bcl-1) - Decreased mortality	(93)
C21	AT_2_R agonist	Curative	MCT rats (4 weeks)	Injection (i.p.), daily, 0.03 mg/kg/day (2 weeks)	- Decreased PAP (↓RVSP) - Decreased RV hypertrophy [↓RV/(LV+S)] - Decreased RV fibrosis (↓ fibrosis area)	(94)
PD-123319	AT_2_R blocker	Curative	MCT rats (4 weeks)	Injection (i.p.) 3 mg·kg^−1^·day (2 weeks)	- Did not decrease PAP (↔RVSP) - Did not decrease RV hypertrophy [↔RV/(LV+S)] - Did not decrease RV fibrosis (↔ fibrosis area)	(94)
A779	Mas antagonist	Curative	MCT rats (4 weeks)	Injection (s.c.) 0.5 mg/kg/day (2 weeks)	- Did not decrease PAP (↔RVSP) - Did not decrease RV remodeling [↔RV/(LV+S)] - Did not decrease RV fibrosis (↔ fibrosis area)	(94)
Macitentan	ET_R_A/ET_R_B blocker	Preventive	PAB rabbits (31 days)	Oral gavage, 10 mg/kg/day (days 1–31)	- Preserved RV function (↑RV S') - Prevented RV cardiomyocyte hypertrophy (↓myocyte size) - Prevented RV fibrosis (↓collagen volume) - Preserved RV gene expressions (↓CTGF, ↑endothelin-1, ↑PDGF, ↑MMP2, ↑MMP9) - Prevented RV apoptosis (↓ TUNEL positive cells, ↓caspase-3, ↓caspase-8)	(64)
SB 217242	ER_R_A blocker	Preventive	HOX rats (14 days)	Osmotic minipump, 10.8 mg/day, (days 0–14)	- Prevented the rise in PAP (↓PAPs) - Did not decrease PA remodeling (↔ wall thickness) - Reduced RV hypertrophy [↓RV/(LV+S)]	(95)
SB 217242	ER_R_A blocker	Curative	HOX rats (28 days)	Osmotic minipump, 10.8 mg/day, (days 14–28)	- Reduced PAP (↓PAPs) - Reduced PA remodeling (↔ wall thickness) - Did not decrease RV hypertrophy [↔RV/(LV+S)]	(95)
A-192621	ET_R_B blocker	Preventive	MCT rats (4 weeks)	Oral gavage, twice daily, 30 mg/kg/d (days 1–28)	- Augmented the increase in PAP (↑RVSP) - Worsened RV hypertrophy [↑RV/(LV+S)] - Did not prevent PA remodeling (↔medial wall thickness)	(96)
ABT-627	ET_R_A blocker	Preventive	MCT rats (4 weeks)	Oral gavage, twice daily, 10 mg/kg/d (days 1–28)	- Prevented the rise in PAP (↓RVSP) - Prevented RV hypertrophy [↓RV/(LV+S)] - Decreased PA remodeling (↔medial wall thickness)	(96)
Bosentan	ET_R_A/ET_R_B blocker	Preventive	MCT rats (4 weeks)	Oral gavage, daily, 100 mg/kg (days 1–28)	- Prevented the rise in PAP (↓RVSP) - Prevented RV hypertrophy [↓RV/(LV+S)] - Prevented PA remodeling (↓medial area)	(97)
Ambrisentan	ET_R_A blocker	Preventive	MCT (4 weeks)	Oral gavage, daily, 35 mg/kg (days 1–28)	- Prevented the rise in PAP (↓RVSP) - Prevented RV hypertrophy [↓RV/(LV+S)] - Decreased PA remodeling (↓medial area)	(97)
LU135252	ET_R_A blocker	Curative	MCT rats (5 weeks)	Chow, 50 mg/kg/d (3 weeks)	- Decreased PAP (↓RVSP) - Did not decrease RV hypertrophy [↔RV/(LV+S)] - Improved RV diastolic function (↓RVEDP)	(98)
BSF420627	ET_R_A/ET_R_B blocker	Curative	MCT rats (5 weeks)	Chow, 50 mg/kg/day (2–3 weeks)	- Decreased PAP (↓RVSP) - Decreased RV hypertrophy [↓RV/(LV+S)] - Improved RV diastolic function (↓RVEDP) - Decreased mortality	(98)
Macitentan	ET_R_A/ET_R_B blocker	Curative	MCT rats (7 weeks)	Oral gavage, daily, 30 mg/kg/day (6 weeks)	- Improved RV function (↑ RVFAC, ↑TAPSE) - Improved RV remodeling (↓RVID, ↓RVWT) - Decreased RV hypertrophy (↓RV/(LV+S) - Decreased RV fibrosis (↓fibrosis area)	(99)
Macitentan	ET_R_A/ET_R_B blocker	Curative	MCT rats (3 weeks)	Chow, 30 mg/kg/day, (20 days)	- Improved RV remodeling (↓RVWT) - Decreased RV fibrosis (↓fibrosis area) - Improved cardiac electrical activity (↓ QT_c_)	(100)
Bosentan	ET_R_A/ET_R_B blocker	Preventive	HOX rats (3 weeks)	Oral gavage, daily (100 mg/kg/day) (3 weeks)	- Did not prevent the rise in PAP (↔RVSP) - Prevented RV hypertrophy (↓RV/(LV+S), ↓RV/BW) - Did not prevent RV wall thickness (↔RVWT) - Did not preserve RV function (↔TAPSE) - Prevented RV fibrosis (↓collagen-1)	(101)
TA-0201	ET_R_A blocker	Preventive	MCT rats (19 days)	Oral gavage, daily (0.5 mg/kg/day) (19 days)	- Prevented the rise in PAP (↓RVSP/LVSP) - Did not prevent RV dilatation (↔RV/LV) - Prevented RV hypertrophy (↓RV/BW)	(102)
Bosentan	ET_R_A/ET_R_B blocker	Curative	MCT rats (25 days)	Oral gavage, daily (100 mg/kg/day) (14–25 days)	- Did not decrease PAP (↔RVSP) - Worsened PVR (↑PVR) - Did not decrease RV dilation (↔RVESD, ↔RVEDD) - Decreased RV function (↓RVFS, ↓CO) - Increased RV cardiomyocyte hypertrophy (↑CSA)	(103)
PD155080	ET_R_A blocker	Preventive	MCT rats (9 weeks)	Chow, 50 mg/kg/day (9 weeks)	- Prevented the rise in PAP (↓RVSP) - Preserved RV diastolic function (↓RVEDP) - Prevented RV hypertrophy (↓RV/BW)	(104)
BMS-193884	ET_R_A blocker	Preventive	MCT rats (20 days)	Chow, 100 mg/kg/day (19 days)	- Prevented the rise in PAP (↓RVSP) - Prevented RV hypertrophy (↓RV weight) - Normalized gene expression (↓ANP)	(105)
Macitentan	ET_R_A/ET_R_B blocker	Curative	SuHx rats (8 weeks)	Oral gavage, 30 mg/kg/day (3 weeks)	- Did not decrease PAP (↔RVSP) - Did not decrease RV hypertrophy [↔RV/(LV+S)] - Improved RV function (↑RVEF) - Reduced PA remodeling - Improved RV metabolism (↓RV FDG uptake)	(106)
Macitentan	ET_R_A/ET_R_B blocker	Preventive	PAB rabbits (6 weeks)	Oral gavage, 20 mg/kg/day (6 weeks)	- Prevented RV fibrosis (↓ fibrosis area) - Prevented RV cardiomyocyte hypertrophy (↓CSA) - Prevented upregulation of proteins driving disease progression (↓CTGF, ↓TGF-β, ↓pSMAD3, pSMAD2) - Prevented the activation of ECM regulators (MMP2 and MMP9) - Prevented RV cardiomyocyte apoptosis (↓TUNEL positive cells) - Preserved RV dysfunction (↓RV S‘, ↓TAPSE, ↓RVFAC, ↑CO) - Preserved RV contractility (↓Ees)	(107)
Macitentan	ET_R_A/ET_R_B blocker	Curative	PAB rabbits (6 weeks)	Oral gavage, 20 mg/kg/day (3 weeks)	- Decreased RV fibrosis (↓ fibrosis area) - Decreased RV cardiomyocyte hypertrophy (↓CSA) - Decreased the expression of proteins driving disease progression (↓CTGF, ↓TGF-β, ↓pSMAD3, pSMAD2) - Decreased the activities of ECM regulators (MMP2 and MMP9) - Decreased RV cardiomyocyte apoptosis (↓TUNEL positive cells) - Preserved RV dysfunction (↓RV S‘, ↓TAPSE, ↓RVFAC, ↑CO) - Preserved RV contractility (↓Ees	(107)
Macitentan	ET_R_A/ET_R_B blocker	Preventive	Bleo rats (4 weeks)	Oral gavage, 100 mg/kg/day (4 weeks)	- Prevented a decrease in RV function (↑RV CO) - Prevented RV hypertrophy [↓RV/(LV+S)] - Prevented RV cardiomyocyte hypertrophy (↓cardiomyocyte diameter) - Prevented PA remodeling (↓pulmonary vascular hypertrophy) - Prevented gene expression changes (↓Col1a1, ↓Fn1, ↓Lgals3, ↓Lox, ↓Nppa, ↓Nppb, ↓timp1, ↓fst, ↓inhba)	(108)
Bosentan	ET_R_A/ET_R_B blocker	Prevented	Bleo rats (4 weeks)	Oral gavage, 300 mg/kg/day (4 weeks)	- Prevented a decrease in RV function (↑RV CO) - Did not prevent RV hypertrophy [↔RV/(LV+S)] - Did note prevent RV cardiomyocyte hypertrophy (↔cardiomyocyte diameter) - Did not prevent PA remodeling (↔pulmonary vascular hypertrophy) - Did not prevent gene expression changes (↔Col1a1, ↔Fn1, ↔Lgals3, ↔Lox, ↔Nppa, ↔Nppb, ↔timp1, ↔fst, ↔inhba)	(108)
Bisoprolol	β1-AR blocker	Curative	MCT rats (31 days)	Oral gavage, daily (10 mg/kg) (10–31 days)	- Did not reduce PAP (↔RVSP) - Improved RV remodeling (↓RVEDD, ↔RVWT) - Improved RV systolic function (↑CO, ↑TAPSE) - Improved RV diastolic function (↓RVEDP) - Improved RV contractility (↑Ees, ↑Ees/Ea) - Reduced RV fibrosis (↓fibrosis area) - Reduced RV inflammation (↓CD45+ cells) - Restored βAR signaling (↑troponin-I phosphorylation, ↑myosin binding protein C phosphorylation)	(109)
Metaprolol	β1-AR blocker	Curative	MCT rats (31 days)	Oral gavage, daily (10 mg/kg) (15–31 days)	- Did not decrease PAP (↔RVSP) - Did not decrease RV hypertrophy [↔RV/(LV+S)] - Improved RV contractility (↑ ESPVR/Ea) - Improved RV cardiomyocyte contractility (↓sarcomere shortening)	(110)
Carvedilol	β1-AR blocker	Curative	SuHx rats (8 weeks)	Oral gavage, daily (15 mg/kg) (4–8 weeks)	- Restored gene expression changes (↑PGC-1α, ↑CD36, ↑ CPT1α, ↑CPT2, ↑ACADM) - Reduced protein degradation system (↓20S proteosome activity, ↓ubiquitinated protein)	(111)
Bisoprolol	β1-AR blocker	Curative	PAB rats (7 weeks)	Oral gavage, 10 mg/kg/day (6 weeks)	- Did not have influence on any of the measured parameters of RV	(90)
Arotinolol	α/β-AR blocker	Preventive	MCT rats (2 weeks)	Osmotic minipump, 0.25 mg/kg/day (2 weeks)	- Prevented the increase in PAP (↓RVSP, ↓sPAP, ↓mPAP, ↓dPAP) - Prevented RV diastolic dysfunction (↓RVEDP) - Prevented RV hypertrophy (↓RV/BW)	(112)
Metoprolol	β1-AR blocker	Curative	MCT rats (21 days)	10 mg/kg1/day (days 14–21)	- Did not decrease PAP (↔RVSP, ↔mPAP) - Decreased PVR (↓PVR) - Improved RV function (↑CO) - Decreased RV hypertrophy [↓RV/(LV+S)] - Decreased PA remodeling (↓PA muscularization)	(113)
Metoprolol	β1-AR blocker	Curative	MCT rats (21 days)	10 mg/kg/day (days 14–21)	- Did not decrease PAP (↔RVSP, ↔mPAP) - Decreased PVR (↓PVR) - Improved RV function (↑CO) - Did not decrease RV hypertrophy [↔RV/(LV+S)] - Decreased PA remodeling (↓PA muscularization)	(113)
Nebivolol	β1-AR blocker	Curative	MCT (21 days)	100 mg/kg1/day1 (days 14–21)	- Did not decrease PAP (↔RVSP, ↔mPAP) - Did not decrease PVR (↔PVR) - Did not improve RV function (↔CO) - Did not decrease RV hypertrophy [↔RV/(LV+S)] - Did not decrease PA remodeling (↔PA muscularization)	(113)
Sarpogrelate	5-HT_2A_R antagonist	Preventive	MCT rats (21 days)	50 mg/kg/day, intraperitoneally (21 days)	- Prevented the rise in PAP (↓mPAP) - Prevented RV hypertrophy [↓RV/(LV+S)] - Prevented PA remodeling (↓medial wall thickness) - Decreased mortality	(114)
Sarpogrelate	5-HT_2A_R antagonist	Curative	MCT rats (6 weeks)	50 mg/kg/day, intraperitoneally (21 days)	- Did not decrease PAP (↔mPAP) - Did not decrease RV hypertrophy [↔RV/(LV+S)] - Did not reduce PA remodeling (↔medial wall thickness)	(114)
Sarpogrelate	5-HT_2A_R antagonist	Preventive	HOX rats (14 days)	Oral gavage, 50 mg/kg/day (14 days)	- Preventive the rise in PAP (↓mPAP) - Prevented RV hypertrophy [↓RV/(LV+S)] - Prevented PA remodeling (↓PA muscularization, ↓medial wall thickness)	(115)
C-122	5-HT_2B_R antagonist	Preventive	MCT rats (21 days)	Oral gavage, 10 mg/kg/day (21 days)	- Prevented the rise in PAP (↓mPAP, ↓sPAP) - Prevented RV hypertrophy (↓RV/BW) - Prevented PA remodeling (↓PA muscularization)	(116)
SB204741	5-HT_2B_R antagonist	Curative	PAB (21 days)	Injection (i.p.), 5 mg/kg/d for	- Did note reduce PAP (↔RVSP) - Decreased RV hypertrophy (↓RV/tibia) - Decreased RV fibrosis (↓total collagen area) - Improved RV function (↑CO)	(117)
Terguride	5-HT_2A_R/5-HT_2B_R antagonist	Curative	PAB (21 days)	Injection (i.p.), 0.2 mg/kg/d	- Did note reduce PAP (↔RVSP) - Decreased RV hypertrophy (↓RV/tibia) - Decreased RV fibrosis (↓total collagen area) - Improved RV function (↑CO)	(117)
Sarpogrelate	5-HT_2A_R antagonist	Preventive	HOX rats (14 days)	Oral gavage, 50 mg/kg/day	- Preventive the rise in PAP (↓mPAP) - Prevented RV hypertrophy (↓RV/TLV+S) - Prevented PA remodeling (↓remodeled vessels)	(118)
GR127935	5-HT_1B/1D_R antagonist	Preventive	HOX rats (14 days)	Oral, 3 mg/kg/day in distilled H_2_O	- Preventive the rise in PAP (↓mRVP) - Prevented RV hypertrophy [↓RV/(LV+S)] - Prevented PA remodeling (↓PA muscularization, ↓medial wall thickness)	(119)
Fluoxetine	5-HTT	Preventive	HOX rats (15 days)	Oral gavage, (10 mg/kg/day)	- Preventive the rise in PAP (↓RVSP) - Prevented RV hypertrophy [↓RV/(LV+S)] - Did not prevent PA remodeling (↓PA muscularization, ↓medial wall thickness)	(120)
Citalopram	5-HTT	Preventive	HOX rats (15 days)	Oral gavage, (10 mg/kg/day)	- Preventive the rise in PAP (↓RVSP) - Prevented RV hypertrophy [↓RV/(LV+S)] - Did not prevent PA remodeling (↔PA muscularization)	(120)
Ketanserin	5-HT_2A_R receptor antagonist	Preventive	HOX rats (14 days)	Injection (i.p.) 2 mg/kg/day	- Did not prevent the rise in PAP (↔RVSP) - Did not prevent RV hypertrophy [↔RV/(LV+S)] - Did not prevent PA remodeling (↔PA muscularization)	(120)
GR127935	5-HT_1B/1D_R antagonist	Preventive	HOX rats (15 days)	Injection (i.p.) 2 mg/kg/day	- Did not prevent the rise in PAP (↔RVSP) - Did not prevent RV hypertrophy [↔RV/(LV+S)] - Did not prevent PA remodeling (↔PA muscularization)	(120)
Treprostinil	Prostanoid	Curative	SuHx rats (7 weeks)	Osmotic minipumps, 100 ng/kg/min (3 weeks)	- Reduced PAP (↓RVSP) - Decreased RV hypertrophy [↓RV/(LV+S)] - Improved RV function (↑CO, ↑TAPSE) - Decreased RV remodeling (↓RVID/LVID, ↓RVWT) - Did not reduce PA remodeling (↔medial wall thickness, ↔occluded vessels)	(121)
Treprostinil	Prostanoid	Preventive	HOX mice (28 days)	Osmotic minipump, 70 ng/kg/min (28 days)	- Prevented PAP increase (↓RVSP) - Did not prevent RV hypertrophy [↔RV/(LV+S)] - Prevented PA remodeling (↓PA muscularization, ↓wall thickness)	(122)
Iloprost	Prostanoid	Curative	MCT rats (42 days)	Nebulization, 6.0 μg/kg/day, 15-min nebulisations were repeated 12 times per day for 2 weeks	- Decreased PAP increase (↓RVSP) - Decreased PA remodeling [↓RV/(LV+S)] - Reduced PVR (↓PVRI) - Decreased PA remodeling (↓PA muscularization, ↓wall thickness)	(123)
Iloprost	Prostanoid	Curative	SuHx rats (6 weeks)	Nebulization, 0.1 μg/kg, 15-min nebulisations were repeated three times daily for 2 weeks	- Decreased PAP (↓mPAP) - Did not decrease RV hypertrophy [↔RV/(LV+S)] - Did not decrease PA remodeling (↔PA muscularization) - Restored RV function (↑CO, ↑TAPSE, ↑running time) - Decreased RV fibrosis (↓ fibrosis area) - Decreased the change of gene expressions (↓CTGF, ↓Cola1a, ↓Cola3, MMP2, MMP9, TIMP2)	(83)
Treprostinil	Prostanoid	Curative	PAB (7 weeks)	Osmotic minipump, 300 ng/kg/minute or 900 ng/kg/minute (6 weeks)	- Did not have effects of any of the measured RV parameters	(124)
Beraprost	Prostanoid	Preventive	MCT rats (19 days)	Oral gavage, daily (100 μg/kg/day) (19 days)	- Prevented the rise in PAP (↓RVSP/LVSP) - Did not prevent RV dilatation (↔RV/LV) - Prevented RV hypertrophy (↓RV/BW)	(102)
Cefminox	IP and PPARγ agonist	Preventive	HOX rats (28 days)	Injection (tail i.v.), 160 mg/kg daily (28 days)	- Prevented the rise in PAP (↓mPAP) - Prevented RV hypertrophy [↓RV/(LV+S)]	(125)
Cefminox	IP and PPARγ agonist	Preventive	HOX rats (28 days)	Injection (tail i.v.), 320 mg/kg daily (days 1–28)	- Prevented the rise in PAP (↓mPAP) - Prevented RV hypertrophy [↓RV/(LV+S)]	(125)
Apelin	Exogenous apelin	Curative	MCT rats (25 days)	Injection (i.p.), daily, 200 μg/kg/day (days 11–24)	- Reduced PAP (↓RVSP) - Reduced RV hypertrophy [↓RV/(LV+S)] - Reduced RV cardiomyocyte hypertrophy (↓cardiomyocyte diameter) - Reduced RV fibrosis (↓fibrosis) - Did not decrease PA remodeling (↔wall thickness) - Normalized gene expressions (↑apelin, ↑APJ, ↓endothelin-1, ↓angiotensin-II, ↑MAS)	(71)

*RVSP, right ventricular systolic pressure; RVEDP, right ventricular end-diastolic pressure; Ees, end-systolic elastance; Ea, arterial elastance; Eed, end-diastolic elastance; PAP, pulmonary artery pressure; mPAP, mean pulmonary artery pressure; sPAP, systolic pulmonary artery pressure; dPAP, diastolic pulmonary artery pressure; BW, body weight; RV, right ventricle; LV, left ventricle; S, septum; PA, pulmonary artery; TAPSE, tricuspid annular plane systolic excursion; PAB, pulmonary artery banding; MCT, monocrotaline; HOX, hypoxia; SuHx, sugen plus hypoxia; Bleo, bleomycin; CO, cardiac output; RVWT, right ventricular wall thickness; RVESD, right ventricular end-systolic diameter; RVEDD, right ventricular end-diastolic diameter; RVID, right ventricular diameter at end-diastole; RVFAC, right ventricular fractional area change; RVEF, right ventricular ejection fraction; RV S', lateral systolic velocity of the tricuspid annulus; PVR, pulmonary vascular resistance; i.p., intraperitoneal; s.c., subcutaneous; CSA, cardiomyocyte cross sectional area*.

### Endothelin Receptors

**Figure 1:**
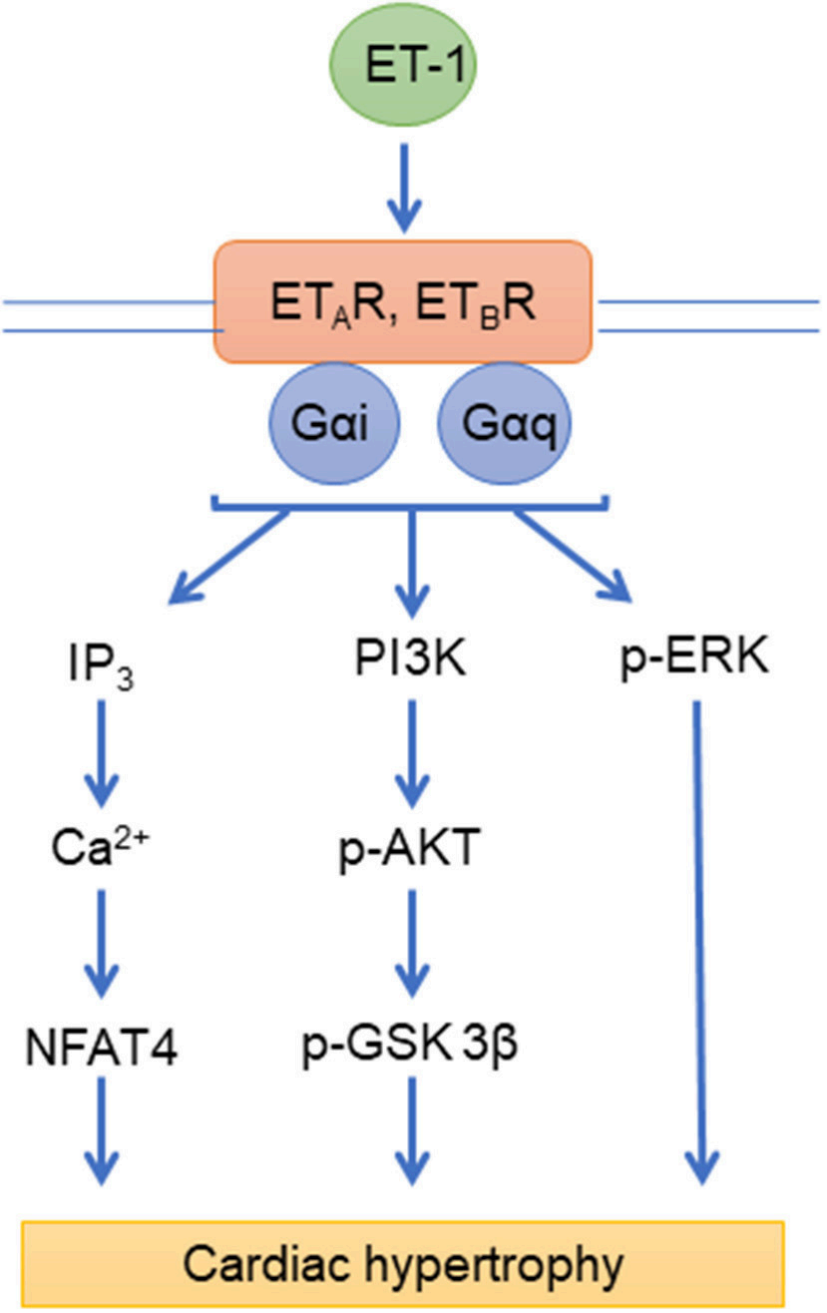
Endothelin signaling pathways. ET_A_R, ET_B_R, endothelin receptors; ET-1, endothelin; IP_3_, inositol 1,4,5-trisphosphate; Ca^2+^, Calcium; NFAT4, nuclear factor of activated T cells 4; PI3K, Phosphatidylinositol-4,5-bisphosphate 3-kinase; p-AKT, phospho-Protein kinase B; p-ERK, phospho- extracellular signal-regulated kinases; Gαi, Gαq, G-proteins.

Endothelin-1 (ET-1) is produced by endothelial cells and acts on pulmonary artery smooth muscle cells (PASMCs) to induce vasoconstriction and cell proliferation, thus actively contributing to the pathogenesis of PAH (126). The effects of ET-1 on target cells are mediated with two distinct GPCRs, the endothelin type A (ET_A_R) and type B (ET_B_R) receptors (127). Notably, these receptors have distinct expression patterns and effects: ET_A_R is expressed primarily on smooth muscle cells and promote vasoconstriction while ET_B_R is expressed primarily on endothelial cells and promotes vasodilation (128). However, the effect of ET-1 is primarily vasoconstriction, as it is the most potent vasoconstrictor in the human cardiovascular system (128). Both ET_A_R and ET_B_R are coupled to Gi and Gq, as well as to β-arrestins (129). This results in the activation of a variety of signaling pathways downstream of the receptor (Figure 1). Activation of endothelin receptors by ET-1 results in the activation of Bcl2, the epidermal growth factor (EGF) receptor (EGF-R) (130), and mitogen-activated protein kinase (PK) cascades (131). These signaling pathways promote cardiomyocyte survival and hypertrophy in response to pressure overload (132). Circulating levels of ET-1 are increased in PAH patients (133, 134) and its levels correlate with pulmonary vascular resistance (PVR), right atrial pressure (RAP) and oxygen saturation in PAH (135). These findings led to the development and subsequent approval of both ET_A_R-specific and dual ET_A/B_R ERAs for the treatment of PAH, including bosentan, ambrisentan, and macitentan (136).

The physiological effects of ERAs are complex and is likely mediated by effects both on the pulmonary circulation and the right ventricle. In monocrotaline (MCT)-induced PH rats, the dual ET receptor antagonist BSF 420627 doubled survival compared to untreated animals and increased survival by 10% compared to the ET_A_-selective antagonist LU 135252 (98). A reduction of RV hypertrophy was only seen in the animals receiving the dual ET receptor antagonist, suggesting that blockade of both ET_A_R and ET_B_R is necessary to prevent all of the deleterious effects of ET-1 in the MCT model. A direct comparison of the dual ET receptor antagonist bosentan and the ET_A_-selective antagonist ambrisentan in the MCT model demonstrated that, compared to bosentan, ambrisentan significantly increased prostacyclin synthase I expression (97). However, both antagonists similarly reduced RV systolic pressure, pulmonary vascular remodeling, and RV hypertrophy. Macitentan, an ET receptor dual antagonist, prevents RV hypertrophy and the development of PH at a dose 10 times lower than bosentan (137), which may simply reflect its higher potency at ET receptors. ET-1 up-regulates HIF-1 alpha, which can contribute to maladaptive remodeling and increased anaerobic metabolism (138). Macitentan treatment reduced PAH severity, lowered RV FDG uptake, and improved RV function in SUHX rats (106). In the Dahl-salt rat model of systemic hypertension, macitentan administered in addition to the maximally effective dose of bosentan further reduced mean arterial pressure (MAP) (97). These differences between ERAs is likely a combination of their different patterns of ET_A/B_R selectivity and different potencies, but some of it may also be due to their selective inhibition of different signaling pathways downstream of these receptors.

However, there are controversies regarding the role of ET-1 in RV failure and only a few studies have addressed this topic. ET-1 and ET_B_R are upregulated in the RV myocardium in MCT-induced RV remodeling in rats (62). This is in contrast to the RV myocardium of PAH patients, where the density of ET_A_R is increased, while ET_B_R is decreased or unchanged (24, 52). Interestingly, while, ET-1 does not influence on contractility and calcium handling of isolated cardiomyocytes from remodeled RV (139), bosentan, an ET_A/B_R antagonist, exerts negative inotropic effect on the hypertrophied RV of MCT rats (24). However, long-term treatment with an ET_A_R antagonist in MCT rats improved RV remodeling due to normalization of calcium handling (104). ET-1 may exert negative inotropic effect on the right ventricle of adults mice (140), or positive inotropic effect on right ventricle of neonatal mice (141) through ET_A_R (140, 141). Thus, the effects of ERAs on the RV are complex and vary depending on the model system used.

### Adrenergic Receptors

**Figure 2:**
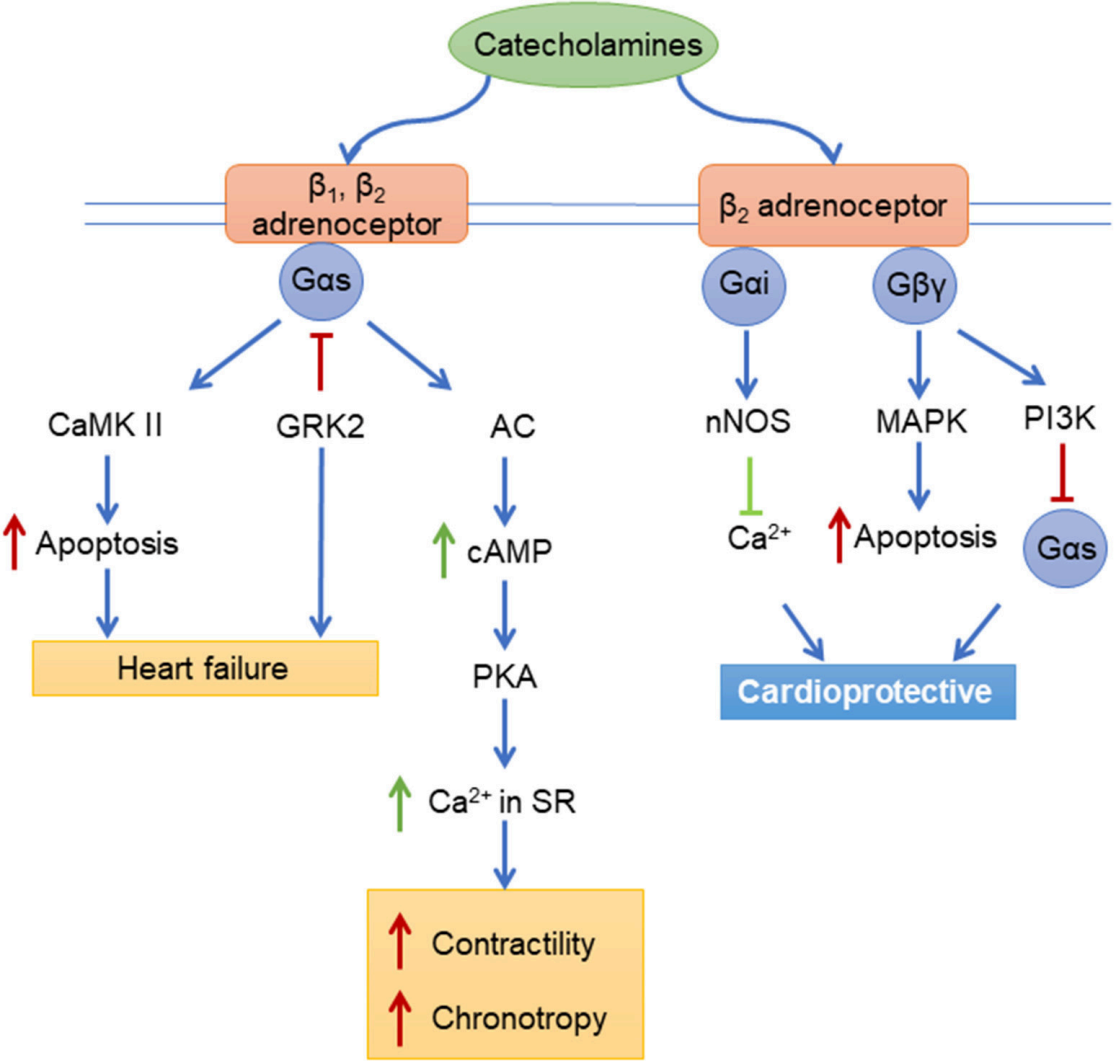
Adrenergic receptors signaling pathways. CaMK II, Ca^2+^/Calmodulin-Dependent Protein Kinase II; GRK2, beta-adrenergic receptor kinase 1; AC, Adenylyl cyclase; cAMP, Cyclic adenosine monophosphate; PKA, protein kinase A; Ca^2+^, Calcium; nNOS, neuronal nitric oxide synthases; MAPK, mitogen-activated protein kinase; P13K, Phosphatidylinositol-4,5-bisphosphate 3-kinase; Gαs, Gαi, Gβγ, G-proteins.

The adrenergic receptors (ARs) are a large family of receptors with three beta (β) ARs (β_1_AR, β_2_AR, and β_3_AR), three alpha (α)_1_ ARs (α_1A_-, α_1B_-, and α_1D_ARs) and three α_2_ARs (α_2A_-, α_2B_-, and α_2C_ARs). The βARs and α_1_ARs are all expressed in the myocardium (142, 143), while the α_2_ARs are expressed in the nervous system. β_1_ and β_2_ARs classically couple to Gs, but can also couple to Gi under certain conditions, while α_1_ARs classically couple to Gq (Figure 2). Both groups of receptors also bind to β-arrestin adapters. These receptors are also tightly regulated by the activity of GRKs, as GRK2 uncouples βAR signaling and inhibition of GRK2 improves RV function in models of right heart failure (144). The development of RV remodeling in response to pressure overload is accompanied by the dysregulation of myocardial adrenergic receptors in several experimental models of RV remodeling in rodents including MCT-induced PAH (54, 55), SU5416-Hypoxia (SuHx)-induced PAH (144), Hypoxia (HOX)-induced PAH (58), and pulmonary artery banding (PAB) (144).

There are interspecies differences in adrenergic signaling changes in response to the pressure overload in the RV myocardium. For example, in a canine model of MCT-induced RV remodeling, RV function is maintained/compensated to the increased pressure overload, which is associated with increased surface expression of both α_1_- and βARs (59). This is in contrast to what is observed in the remodeled RV myocardium in MCT rats (54, 56–58) and HOX rats (58), where the surface expression of both β_1_AR and β_2_AR are decreased. This finding is likely related to sympathetic hyperactivity and subsequent downregulation of adrenergic receptors in the RV myocardium (145), which is also observed clinically in PAH (146) and in other preclinical disease models such as HOX rats (145). Similarly, MCT rats have increased levels of plasma norepinephrine along with increased content of both epinephrine and norepinephrine in the remodeled RV tissue (147). Moreover, plasma levels of norepinephrine in PAH patients with severe RV failure are correlated with the parameters of pulmonary hemodynamics and cardiac function (135).

For many years in left heart failure, it was unclear as to whether to treat with βAR agonists or β-adrenergic blockers (beta-blockers) until the discovery that beta-blockers improved mortality in chronic systolic heart failure by improving βAR expression on cardiomyocytes (148). At this time, it is unclear as to whether targeting the RV with beta-blockers will have similar protection in the setting of high afterload in PAH. This equipoise has encouraged scientists to perform studies evaluating the effects of both βAR agonists and beta-blockers on pulmonary hemodynamics and RV function in different animal models. Carvedilol, a non-selective beta-blocker targeting β1-AR, β2-AR, and α1-AR, improves RV function and fibrosis without effecting on pulmonary vasculature in MCT-treated rats (149) as well as in SuHx rats (150). The beneficial effect of carvedilol is mediated through the modulation of TGFβ1-CTGF signaling (149) as well as signaling pathways involved in cardiac hypertrophy, protein ubiquitination and mitochondrial function (111). Similarly, another beta-blocker, metoprolol improves the remodeling and function of the pressure overloaded RV in MCT rats (110, 151), mainly by improving RV metabolism (110) and calcium handling (151). In contrast, bisoprolol does not exert beneficial effects on the RV in PAB-operated rats (90). In line with this research, treatment with pyridostigmine (PYR), an oral acetylcholinesterase inhibitor, an activator of parasympathetic system, in the SuHx rats, delays progression to RV failure and improves load-independent indices of RV function mainly due to decreased RV inflammation through the reduced leukocyte infiltration and reduced indices of pulmonary vascular remodeling (53). Interestingly, the density of muscarinic acetylcholine receptors, another GPCR, is not changed in RV remodeling in MCT rats (57). In addition, the effect of adrenergic signaling on cardiac function changes depending on whether RV is remodeled or not. For example, activation of α_1_AR causes negative inotropic healthy RV, while in the failing RV myocardium, stimulation of α_1_AR exerts positive inotropic effect (152, 153). Following this findings, a recent study showed that a selective α_1_AR A type agonist A61603 ameliorates RV remodeling in bleomycin-induced RV remodeling by improving RV antioxidant system and RV fibrosis (154).

### Serotonin Receptors

PAH can be caused by exposure to specific drugs, and serotonin 5-HT_2B_ agonists (155), such as Fen-Phen, have a “definite” association with the development of PAH (136). Consistent with this, disturbed serotonin metabolism contributes to the development and progression of PAH (156) and antagonists of serotonin receptors are beneficial in the preclinical models of PAH (157). There are multiple serotonin receptors, including the 5-HT_1A,B,D,E,F_ (which couple to Gi), 5-HT_2A,B,C_ (which couple to Gq), and the 5-HT_4,6,7_ (which couple to Gs) (129). Many of these receptor subtypes are expressed in the RV and pulmonary circulation (21). PAH patients display increased levels of circulating serotonin (158, 159). Serotonin effects on the target cells using its GPCRs and some of its receptors have been found to be upregulated in the remodeled pulmonary arteries. Several serotonin receptor antagonists have been studied in animal models of PAH and RV remodeling and some of them have been found to be efficacious to reverse or prevent the disease (114–120). However, little is known about the effect of serotonin on the RV remodeling and only few studies have focused specifically on the RV using PAB models. The expression of serotonin receptor 5-HT_2B_R is increased in the remodeled RV myocardium in PAB-operated mice (117) and treatment with serotonin receptor antagonists terguride or SB204741 reduce RV fibrosis and improve RV function in PAB-operated mice, a beneficial effect mediated through diminished TGF-β1 induced collagen synthesis by RV cardiac fibroblasts (117). Moreover, the serotonin system works in concert with adrenergic and angiotensin systems to induce cardiac hypertrophy (160).

### Prostanoid Receptors

**Figure 3:**
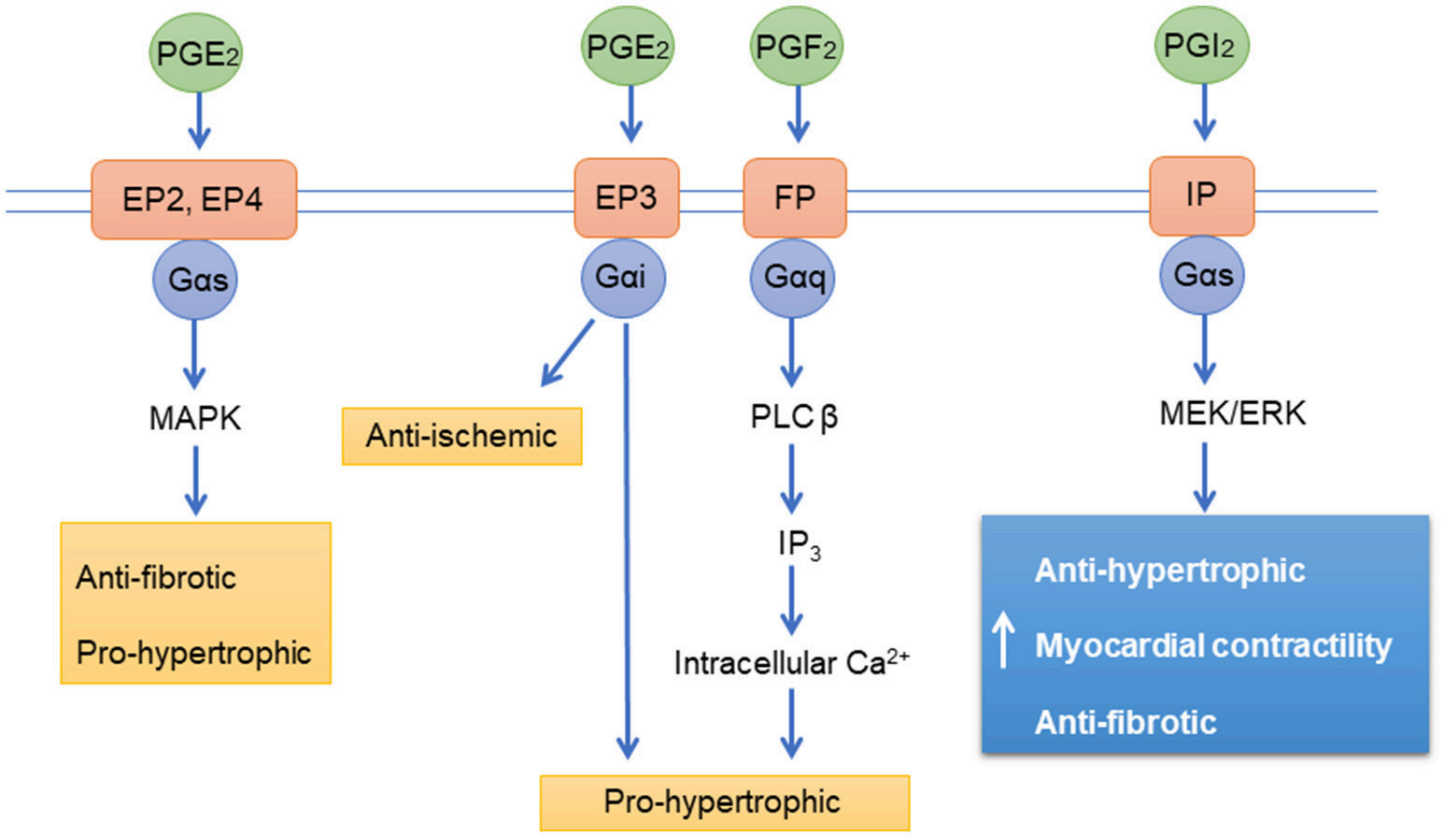
Prostanoid receptors signaling pathways. PGE_2_, Prostaglandin E2; PGF_2_, Prostaglandin F2; PGI_2_, Prostaglandin I2 (Prostacyclin); EP, Prostaglandin receptors; FP, Prostanoid receptors; IP, Prostacyclin receptor; PLC β, 1-Phosphatidylinositol-4,5-bisphosphate phosphodiesterase beta-1; ERK, extracellular signal-regulated kinases IP3, inositol 1,4,5-trisphosphate; Ca^2+^, Calcium; MAPK, mitogen-activated protein kinase; Gαs, Gαi, Gαq, G-proteins.

Prostanoids are a group of lipid-based molecules that modulate vascular tone, platelet function, inflammation, cell proliferation and cardiac function (161). Prostanoids exerts their effects with GPCR prostanoid receptors including DP_1−2_, EP_1−4_, FD, IP, and TP (162) and majority of them are present on cardiomyocytes (163). The prostacyclin receptor (IP) is abundantly expressed in blood vessels, leukocytes, and platelets, and is activated by binding of the prostacyclin and its analogs. IP receptors are coupled to Gs and Gq proteins (Figure 3). The activated IP stimulates adenyl cyclase activity via Gs proteins, increasing cAMP levels in the cells. IP can also activate vasoconstrictive pathways via Gq coupling under certain circumstances (164, 165). The ligands for IP receptors (prostacyclin and its analogs) also bind and activate EP receptors (166); these receptors are not only expressed on the cell membrane but also in the nucleus (167, 168). IP receptor activation leads to the activation of peroxisome proliferator-activated receptor alpha and delta (PPARα and PPARδ) via IP receptor-dependent PKA activation (169). The enzyme prostaglandin-I synthase (PGI) produces prostacyclin, which can activate apoptosis through PPARδ (170). There is evidence that PPARδ is also involved in the acute signaling in prostacyclin-induced vasodilatation (171).

Several synthetic prostanoids have been developed and approved for the treatment of PAH including epoprostenol (IP receptor agonist), treprostinil (IP and EP2 receptor agonist), and iloprost (IP, EP1, EP3, and EP4 receptor agonist) (162). Although, prostanoids reverse/prevent pulmonary artery remodeling and pulmonary hemodynamics in a number of animal models of PAH including MCT, SuHx, and HOX rats (172), only few studies have specifically focused on the effect of prostanoids on RV function. For instance, in MCT and aortocaval-shunting models of RV remodeling, iloprost treatment improves RV capillary-to-myocyte ratio and RV fibrosis with no effect on pulmonary hemodynamics (173). Similarly, treatment with inhaled iloprost of SuHx rats, improves RV function and exercise performance without influencing on RV pressure overload, RV hypertrophy, RV capillarization, and PA remodeling (83). In addition, inhaled iloprost treatment of PAB-operated rats normalizes the expressions of ECM components in RV myocardium (83). The cardiac effect of prostanoids may have chamber specific effects on cardiomyocyte contractility as it was shown that beraprost, a synthetic prostanoid does not influence on RV cardiomyocyte contractility, while increasing the contractility of atrial cardiomyocytes (174).

### The Angiotensin System

**Figure 4:**
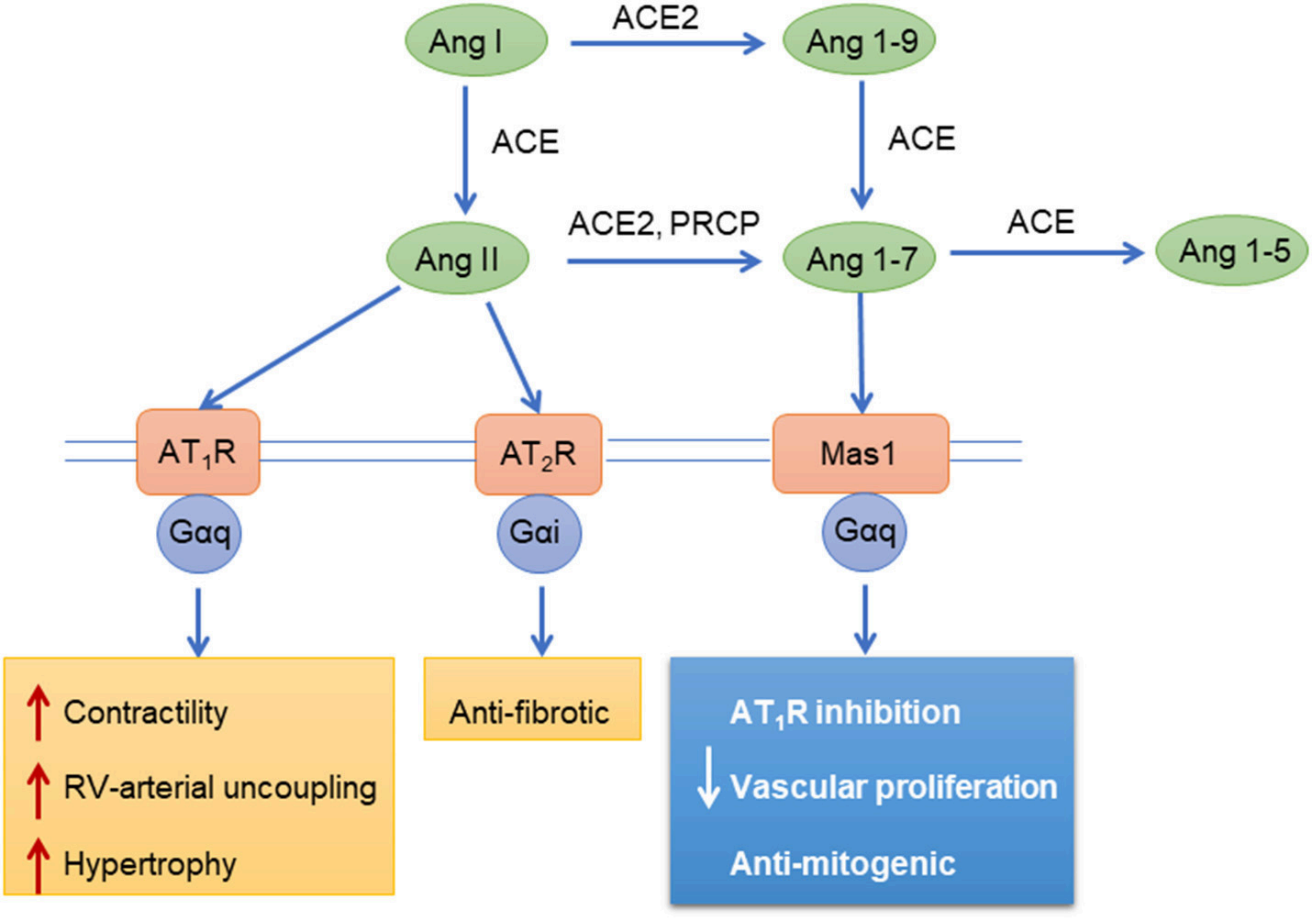
Angiotensin mediated signaling pathways. Ang, angiotensin; ACE, Angiotensin-converting enzyme; AT_1_R, Angiotensin II receptor type 1; AT_2_R, Angiotensin II receptor type 2; Mas1, Proto-oncogene Mas; PRCP, Lysosomal Pro-X carboxypeptidase; Gαi, Gαq, G-proteins.

Both right and left heart failure is associated with neurohormonal activation of the renin-angiotensin-aldosterone system (RAAS), which is associated with disease progression and prognosis in PAH (91). Pulmonary endothelial cells are a rich source of angiotensin converting enzyme (ACE), which converts angiotensin I (Ang-I) to angiotensin-II (Ang-II) (175). Ang-II exerts its effects on target cells with two subtypes of angiotensin GPCRs, Ang-II type 1 receptor (AT_1_R) and Ang-II type 2 receptor (AT_2_R). These receptors have distinct effects, as the AT_1_R promotes vasoconstriction through Gq/11 while AT_2_R promotes vasodilation through Gi (Figure 4). The main effect of Ang-II physiologically is proliferation, hypertrophy, migration, and vasoconstriction of vascular cells through AT_1_R, which promotes pulmonary vascular and RV remodeling. Through the AT_1_R, Ang-II activates mitogen-activated protein kinases (MAPK), receptor tyrosine kinases (RTK), and non-receptor tyrosine kinases. Ang-II also promotes hypoxia inducible factor-1α (HIF-1α) accumulation and activates cyclin-dependent kinase p27 (Kip1) to promote cell hypertrophy and increased oxidative stress (176, 177) through reactive oxygen species generated by NADPH oxidase, which leads to vasoconstriction and inflammation (178). Increased ACE activity and Ang-II production augments pulmonary smooth muscle cell proliferation through AT_1_R signaling (91, 179). Evidence suggests that RAAS is involved in the progression of pulmonary artery remodeling, and agents that inhibit RAAS are beneficial for the RV to cope better with the pressure overload (180).

AT_1_R and AT_2_R are upregulated in the RV myocardium in several animal models of RV remodeling including in MCT rats (55). Receptor expression changes over the course of hypoxia exposure in rats, with increased AT_1_R lower AT_2_R expression in the initial stages (65). However, there are studies indicating that expression of Ang-II receptors in the RV tissue are not changed in MCT rats (65) or even downregulated in PAB rats (68). Moreover, the activity and expression of ACE are increased in the fibrotic areas of the RV myocardium in HOX rats (79) suggesting its involvement in RV remodeling. In a rabbit PAB model, Ang-II increased RV collagen volume to ~3-fold and increased expression of the profibrotic mediators transforming growth factor-β1, connective tissue growth factor, and ET-1 were noticed in this model (181). However, cardiomyocyte specific angiotensinogen overexpressing mice spontaneously develop RV and LV hypertrophy without cardiac fibrosis (182).

Multiple studies have tested the effects of ACE-1 inhibitors and Ang-II receptor blockers on the RV in models of right heart failure and hypertrophy. Several Ang-II receptor blockers including losartan (70, 90, 91), candesartan (80), telmisartan (92), and PD-123319 (94) have been studied in several animal models of RV remodeling such as MCT rats (91–94), PAB rats (90), PAB rabbits (70), and PAB dogs (80). The majority of these agents have demonstrated beneficial effects of RAAS inhibition on RV remodeling and function in several models (70, 80, 91–94). However, a lack of effect of RAAS inhibition on the RV also has been reported (90). In addition, in preclinical studies, inhibition ACE-1 activity with enalapril (75, 89), captopril (87, 88), or ramipril (70) delivered direct beneficial effect on the RV without reducing PAP. Consistent with this, renal denervation (modulating both sympathetic and RAAS activity) improves pulmonary hemodynamics along with attenuation of RV fibrosis and diastolic stiffness (183). However, the aldosterone antagonist eplerenone does not exert beneficial effects on the RV in PAB models in mice (184) and rats (68).

Alternative processing of angiotensin yields peptides that have opposing effects to Ang-II. Angiotensin converting enzyme-2 (ACE2) cleaves Ang-I and Ang-II to yield angiotensin-(1–7) [Ang-(1–7)], angiotensin-(1–9), and angiotensin-(1–5) (185, 186). These peptides reduce pulmonary vascular and RV remodeling through the Mas receptor and AT_2_R in PAH (187–189) (Figure 4). In pulmonary vascular and RV remodeling, cell proliferation, hypertrophy and pro-fibrotic signaling pathways are inhibited by ACE2/Ang-(1–7)/Mas receptor activation (190). Also, ACE2 inhibits ERK 1/2 and JAK2-STAT3 signaling, thereby reducing PASMC proliferation and migration (191). ACE2/Ang-(1–7) has also been shown to decrease cellular oxidant stress through downregulation of NADPH oxidase and improves pulmonary NO synthesis (190). ACE2/Ang-(1–7) exert antifibrotic effects by reducing oxidant stress, transforming growth factor-β levels and collagen production (190). In the RV, ACE2/Ang-(1–7) maintains NO levels, enhance cardiomyocyte calcium handling and improve myocardial contractility (186). Therefore, ACE2/Ang-(1–7)/Mas signaling holds therapeutic potential in RV and PAH.

### Chemokine Receptors

Increased circulating levels of several chemokines have been observed in PAH patients including CXCL10 (192–194), CXCL12 (193), CXCL13 (195), and CXCL16 (193) and some of them were correlated with NT-pro-BNP and the parameters of the RV function such as TAPSE and RV EF (193). Since, chemokines have diverse biological functions, some of them may be beneficial in PAH, as it was shown that elevated levels of CXCL10 are associated with improved survival of patients (192). Notably, CXCL10 decreases proteoglycan synthesis by cardiac fibroblasts (73) thus potentially improving the remodeling extracellular matrix. However, some chemokines may simply be bystanders with no direct effect on the pulmonary vasculature or RV, or may have a yet-discovered role in PAH. For instance, despite of increased circulating levels of CXCL13 in PAH and CTEPH patients, its levels are not correlated with disease severity and outcome (195). Apart being expressed in the pulmonary vasculature, the expressions of several chemokines have been increased in the RV myocardium in both acute (74, 84, 85, 196) and chronic RV failure (73).

The chemokine expressions in the RV myocardium may be independent of pulmonary vasculature and be solely driven by excessive mechanical stress imposed on the RV wall. In a mouse model of PAB-induced RV remodeling, several members of the chemokines have been upregulated in the RV myocardium, including CCL2, CCL5, CXCL16, CXCL10, and CX3CL1 (73). Moreover, acute RV remodeling in pulmonary embolism models in rats are also associated the upregulation of several chemokines including CC-chemokines (CCL2, 3, 4, 6, 7, 9, 17, 20, 27) and CXC-chemokine genes (CXCL1, 2, 9, 10, 16) (74, 84, 85). Similarly, in acute RV remodeling in PAB pigs CCL2, CXCL6, and CXCL2 chemokines are upregulated (86). However, other chemokines such as XCL-1 and CXCL-12 are downregulated in the acutely remodeled RV myocardium (74). The detrimental effect of upregulated chemokines on the RV may be due to their contribution to the cardiac fibrosis mediated by the upregulation of several proteoglycans by cardiac fibroblasts (73, 197). However, the roles of chemokine receptors have not been studied specifically in RV remodeling models and only few studies showed that some of them are upregulated in the remodeled RV myocardium such as LCR1 in HOX mice (60), CCR2 in PAB mice (73), and CCR1 and CXCR4 in rat model PE (74).

### Apelin Receptor

Apelin and elabela/toddler are endogenous ligands for the apelin receptor APJ, which has been shown to play a beneficial role in normal physiology and its dysregulation is associated with several cardiopulmonary diseases (198), including PAH (199). Depending on the disease model and species used, apelin expression in the lung tissue has been noted unchanged (81), or upregulated in HOX mice (200). Nevertheless, apelin-KO mice develop more severe PAH upon exposure to hypoxia (200) suggesting the beneficial role apelin in PAH. In line with findings of preclinical studies, circulating levels of apelin are decreased in PAH patients (200), which may be due to decreased expression of apelin in endothelial cells of remodeled pulmonary arteries (201, 202). Moreover, in endothelial cell specific PPAR-γ deficient mice, which spontaneously develop pulmonary hypertension, treatment with apelin reverses PAH (201). RV myocardial expressions of apelin and its receptor APJ are dysregulated differently depending on the severity of pressure overload imposed on the RV wall. Apelin and its receptor are downregulated in maladaptive RV remodeling models such as SuHx rats (82), MCT rats (71, 72) while their expression is increased in adaptive RV remodeling models such as HOX rats (81). Moreover, treatment with pyroglutamylated apelin-13 (200 μg/kg/day, ip) of MCT rats attenuates RV cardiomyocyte hypertrophy and RV fibrosis along with restoration of apelin-AJP signaling in the RV without effecting on PA remodeling (71). Similarly, treatment with Elabela/Toddler, an endogenous analog of apelin, of MCT rats attenuates RV hypertrophy and PA remodeling (72). Importantly, similar to apelin, elabela/toddler exerts positive inotropic effects on both LV and RV (72). These findings have led to clinical trials with APJ agonists as a treatment option for PAH. For instance, in patients with PAH, 5-min intravenous infusions of increasing doses of (Pyr1) apelin-13 at 10, 30, and 100 nmol/min reduced PVR and increase CO without effecting on systemic hemodynamics (203).

### Human Studies With GPCR Agonists and Antagonists

We refer readers to the following excellent review articles summarizing clinical trials with GPCR agonists or/and antagonists in PH patients including endothelin receptor antagonists (204), prostacyclin receptors agonists (205), and beta-blockers (206). Here we briefly discuss clinical studies evaluating the effects of above-mentioned drugs on the RV in PH patients. As discussed above, promising effects of GPCR agonists/antagonists on the pressure-overloaded RV in animal studies have led to several clinical trials focusing not only on pulmonary hemodynamics but also on RV performance in PH patients.

Recent trials showed that initial upfront combination treatment with ERAs and PDE5 inhibitors improved RV remodeling and function in patients with scleroderma associated PAH (207) as well as in IPAH patients (208). Similar to animal studies, adrenergic receptors of the in the RV in human PAH are dysregulated (144, 209, 210). Despite the beneficial effects of beta-blockers in some models of PAH and RV remodeling, it is still unclear as to the potential beneficial effects of these drugs on the RV in patients with PAH. Bisoprolol (a selective β1AR-blocker) treatment for 6 months in 18 IPAH patients was associated with a reduced cardiac output and a trend toward reduced 6-min walk distance (211). Another study showed that in PH patients with different etiologies, carvedilol treatment was well tolerated and associated with maintenance of cardiac output and no improvement in 6-min walk distance with beneficial effect on RV metabolism (212).

Initially, the cardiac effects of prostanoids were studied in patients with heart failure and were found to be beneficial in these patients (213). Similarly, in PAH patients, prostanoids improve RV performance, functional and hemodynamic outcomes, and survival (214–218). A meta-analysis revealed that despite the fact that all forms of prostanoids improve hemodynamics and functional outcomes, only intravenous prostanoids provide significant survival benefit in PAH (218). However, in patients with left heart failure, treatment with epoprostenol is associated with increased mortality at 6 months, despite an early improvement in exercise capacity (219). Some have speculated that these early improvements in exercise performance and cardiac output after prostanoid therapy may be due to increased RV contractility, which subsequently may lead to increased myocardial oxygen consumption and may therefore be detrimental (220). Recently in a study of PAH patients, treprostinil treatment was associated with a decrease in afterload with no increase in inotropy (221). Therefore, it is still unclear as to the degree of which a direct effect of prostacyclins on the RV plays in the treatment of PAH.

One of the consequences of activated RAAS is increased levels of circulating aldosterone in PAH patients (222). In PAH patients, combination treatment with an aldosterone inhibitor, spironolactone and an ERA, ambrisentan lead to more significant improvements in functional status and cardiac performance compared to ambrisentan alone (223). The results of a randomized controlled trial (Clinicaltrials.gov NCT01468571) evaluating the safety and tolerability in PAH patients showed that it is safe and well tolerated (224). Another trial assessing the effects of the aldosterone inhibitor spironolactone in PAH is expected to complete in 2021 (Clinicaltrials.gov NCT01712620). In addition, clinical trials have shown that the use of agents modulating RAAS does not seem to be beneficial for the right ventricle of patients with congenital heart diseases (225). Taken together, despite the well-established beneficial effects of some of the GPCR agonists and antagonists on pulmonary hemodynamics in PAH, their direct effects on the RV are still somewhat controversial and require further study.

## Conclusions

A number of GPCRs are differentially regulated in the RV myocardium in response to pressure overload in both PAH patients and preclinical models of RV remodeling (Table 1). In addition, levels of endogenous ligands targeting GPCRs are changed in the remodeled RV myocardium (Table 2). In preclinical studies of RV failure, some pharmacological agents targeting GPCRs have been shown to be beneficial while others do not appear to have any effects or are even detrimental (Table 3). However, the majority of preclinical studies have been performed using afterload-dependent models such as MCT-, hypoxia-, and SuHx-induced PAH models in which any changes in RV function are confounded by changes in the pulmonary vasculature. This is not true in PAB models, which allow the study of GPCRs and their endogenous and exogenous agonists or antagonists independent from direct effects on the pulmonary vasculature; however, such models have rarely been used. Taken together, the evidence is clear that several GPCRs are dysregulated in the RV myocardium in response to pressure overload are associated with RV remodeling and dysfunction. However, the underlying mechanisms that underlie GPCR function in the RV have not been fully elucidated, which must be addressed in future studies which could lead to novel therapies for right heart failure.

## Author Contributions

SR conceived the review. GV and AM drafted the manuscript. GV, AM, RS, and SR revised the manuscript critically for important intellectual content. RS and SR approved the final version of the manuscript submitted.

### Conflict of Interest Statement

The authors declare that the research was conducted in the absence of any commercial or financial relationships that could be construed as a potential conflict of interest.

## References

[1] Baggen VJ Leiner T Post MC Van Dijk AP Roos-Hesselink JW Boersma E. Cardiac magnetic resonance findings predicting mortality in patients with pulmonary arterial hypertension: a systematic review and meta-analysis. Eur Radiol. (2016) 26:3771–80. 10.1007/s00330-016-4217-6 26847041 PMC5052291

[2] Kjaergaard J Akkan D Iversen KK Køber L Torp-Pedersen C Hassager C. Right ventricular dysfunction as an independent predictor of short-and long-term mortality in patients with heart failure. Eur J Heart Fail. (2007) 9:610–6. 10.1016/j.ejheart.2007.03.001 17462946

[3] Iglesias-Garriz I Olalla-Gomez C Garrote C Lopez-Benito M Martin J Alonso D. Contribution of right ventricular dysfunction to heart failure mortality: a meta-analysis. Rev Cardiovasc Med. (2012) 13:e62–9. 23160163 10.3909/ricm0602

[4] Gorter TM Hoendermis ES Van Veldhuisen DJ Voors AA Lam CS Geelhoed B. Right ventricular dysfunction in heart failure with preserved ejection fraction: a systematic review and meta-analysis. Eur J Heart Fail. (2016) 18:1472–87. 10.1002/ejhf.630 27650220

[5] Burgess MI Mogulkoc N Bright-Thomas RJ Bishop P Egan JJ Ray SG. Comparison of echocardiographic markers of right ventricular function in determining prognosis in chronic pulmonary disease. J Am Soc Echocardiogr. (2002) 15:633–9. 10.1067/mje.2002.118526 12050605

[6] Chang CL Robinson SC Mills GD Sullivan GD Karalus NC Mclachlan JD. Biochemical markers of cardiac dysfunction predict mortality in acute exacerbations of COPD. Thorax (2011) 66:764–8. 10.1136/thx.2010.155333 21474497

[7] Bogaard HJ Abe K Noordegraaf AV Voelkel NF. The right ventricle under pressure: cellular and molecular mechanisms of right-heart failure in pulmonary hypertension. Chest (2009) 135:794–804. 10.1378/chest.08-0492 19265089

[8] Luitel H Sydykov A Schymura Y Mamazhakypov A Janssen W Pradhan K. Pressure overload leads to an increased accumulation and activity of mast cells in the right ventricle. Physiol Rep. (2017) 5:e13146. 10.14814/phy2.13146 28330950 PMC5371552

[9] Vonk-Noordegraaf A Haddad F Chin KM Forfia PR Kawut SM Lumens J. Right heart adaptation to pulmonary arterial hypertension: physiology and pathobiology. J Am Coll Cardiol. (2013) 62:D22–33. 10.1016/j.jacc.2013.10.027 24355638

[10] Frump AL Bonnet S De Jesus Perez VA Lahm T. Emerging role of angiogenesis in adaptive and maladaptive right ventricular remodeling in pulmonary hypertension. Am J Physiol Lung Cell Mol Physiol. (2017) 314:L443–460. 10.1152/ajplung.00374.2017 29097426 PMC5900357

[11] Sydykov A Mamazhakypov A Petrovic A Kosanovic D Sarybaev AS Weissmann N. Inflammatory mediators drive adverse right ventricular remodeling and dysfunction and serve as potential biomarkers. Front Physiol. (2018) 9:609. 10.3389/fphys.2018.00609 29875701 PMC5974151

[12] Egemnazarov B Crnkovic S Nagy BM Olschewski H Kwapiszewska G Right ventricular fibrosis and dysfunction: actual concepts and common misconceptions. Matrix Biol. (2018) 68–69: 507–21. 10.1016/j.matbio.2018.01.010 29343458

[13] McLaughlin VV Presberg KW Doyle RL Abman SH Mccrory DC Fortin T. Prognosis of pulmonary arterial hypertension: ACCP evidence-based clinical practice guidelines. Chest (2004) 126:78S–92S. 10.1378/chest.126.1_suppl.78S 15249497

[14] Hopkins WE Ochoa LL Richardson GW Trulock EP Comparison of the hemodynamics and survival of adults with severe primary pulmonary hypertension or Eisenmenger syndrome. J Heart Lung Transplant. (1996) 15:100–5. 8820089

[15] Hurdman J Condliffe R Elliot C Davies C Hill C Wild J. ASPIRE registry: assessing the Spectrum of Pulmonary hypertension Identified at a REferral centre. Eur Respir J. (2011) 39: 945–55. 10.1183/09031936.00078411 21885399

[16] Gomez-Arroyo J Santos-Martinez LE Aranda A Pulido T Beltran M Muñoz-Castellanos L. Differences in right ventricular remodeling secondary to pressure overload in patients with pulmonary hypertension. Am J Respir Crit Care Med. (2014) 189:603–6. 10.1164/rccm.201309-1711LE 24579837

[17] Westerhof BE Saouti N Van Der Laarse WJ Westerhof N Vonk Noordegraaf A. Treatment strategies for the right heart in pulmonary hypertension. Cardiovasc Res. (2017) 113:1465–73. 10.1093/cvr/cvx148 28957540 PMC5852547

[18] Yancy CW Jessup M Bozkurt B Butler J Casey DE Jr Drazner MH. 2013 ACCF/AHA guideline for the management of heart failure: a report of the American College of Cardiology Foundation/American Heart Association Task Force on Practice Guidelines. J Am Coll Cardiol. (2013) 62:e147–239. 10.1161/CIR.0b013e31829e8776 23747642

[19] Hauser AS Attwood MM Rask-Andersen M Schiöth HB Gloriam DE. Trends in GPCR drug discovery: new agents, targets and indications. Nat Rev Drug Discov. (2017) 16:829. 10.1038/nrd.2017.178 29075003 PMC6882681

[20] Belmonte SL Blaxall BC. Conducting the G-protein Coupled Receptor (GPCR) signaling symphony in cardiovascular diseases: new therapeutic approaches. Drug Discov Today Dis Models (2012) 9:e85–90. 10.1016/j.ddmod.2012.03.001 23162605 PMC3496286

[21] Iyinikkel J Murray F. GPCRs in pulmonary arterial hypertension: tipping the balance. Br J Pharmacol. (2018) 175:3063–79. 10.1111/bph.14172 29468655 PMC6031878

[22] Galie N Manes A Negro L Palazzini M Bacchi-Reggiani ML Branzi A. A meta-analysis of randomized controlled trials in pulmonary arterial hypertension. Eur Heart J. (2009) 30:394–403. 10.1093/eurheartj/ehp022 19155250 PMC2642921

[23] Handoko M De Man F Allaart C Paulus W Westerhof N Vonk-Noordegraaf A. Perspectives on novel therapeutic strategies for right heart failure in pulmonary arterial hypertension: lessons from the left heart. Eur Respir Rev. (2010) 19:72–82. 10.1183/09059180.00007109 20956170 PMC9491638

[24] Nagendran J Sutendra G Paterson I Champion HC Webster L Chiu B. Endothelin axis is upregulated in human and rat right ventricular hypertrophy. Circ Res. (2013) 112:347–54. 10.1161/CIRCRESAHA.111.300448 23233754

[25] Holmboe S Andersen A Johnsen J Nielsen JM Nørregaard R Bøtker HE. Inotropic effects of prostacyclins on the right ventricle are abolished in isolated rat hearts with right-ventricular hypertrophy and failure. J Cardiovasc Pharmacol. (2017) 69:1–12. 10.1097/FJC.0000000000000435 27652910

[26] Michelakis ED. Response to lazarus. Circ Res. (2014) 114:e31. 10.1161/CIRCRESAHA.114.303719 24625731

[27] Van De Veerdonk MC Kind T Marcus JT Mauritz G-J Heymans MW Bogaard H-J. Progressive right ventricular dysfunction in patients with pulmonary arterial hypertension responding to therapy. J Am Coll Cardiol. (2011) 58:2511–9. 10.1016/j.jacc.2011.06.068 22133851

[28] Lagerstrom MC Schioth HB. Structural diversity of G protein-coupled receptors and significance for drug discovery. Nat Rev Drug Discov. (2008) 7:339–57. 10.1038/nrd2518 18382464

[29] Smith JS Rajagopal S. The beta-arrestins: multifunctional regulators of G protein-coupled receptors. J Biol Chem. (2016) 291:8969–77. 10.1074/jbc.R115.713313 26984408 PMC4861465

[30] Benovic JL Strasser RH Caron MG Lefkowitz RJ. Beta-adrenergic receptor kinase: identification of a novel protein kinase that phosphorylates the agonist-occupied form of the receptor. Proc Natl Acad Sci USA. (1986) 83:2797–801. 10.1073/pnas.83.9.2797 2871555 PMC323393

[31] Lohse MJ Benovic JL Codina J Caron MG Lefkowitz RJ. Beta-Arrestin: a protein that regulates beta-adrenergic receptor function. Science (1990) 248:1547–50. 10.1126/science.2163110 2163110

[32] Goodman OB Jr Krupnick JG Santini F Gurevich VV Penn RB Gagnon AW. Beta-arrestin acts as a clathrin adaptor in endocytosis of the beta2-adrenergic receptor. Nature (1996) 383:447–50. 10.1038/383447a0 8837779

[33] Oakley RH Laporte SA Holt JA Barak LS Caron MG. Association of beta-arrestin with G protein-coupled receptors during clathrin-mediated endocytosis dictates the profile of receptor resensitization. J Biol Chem. (1999) 274:32248–57. 10.1074/jbc.274.45.32248 10542263

[34] Laporte SA Oakley RH Holt JA Barak LS Caron MG. The interaction of beta-arrestin with the AP-2 adaptor is required for the clustering of beta 2-adrenergic receptor into clathrin-coated pits. J Biol Chem. (2000) 275:23120–6. 10.1074/jbc.M002581200 10770944

[35] Luttrell LM Ferguson SS Daaka Y Miller WE Maudsley S Della Rocca GJ. Beta-arrestin-dependent formation of beta2 adrenergic receptor-Src protein kinase complexes. Science (1999) 283:655–61. 10.1126/science.283.5402.655 9924018

[36] Gao H Sun Y Wu Y Luan B Wang Y Qu B. Identification of beta-arrestin2 as a G protein-coupled receptor-stimulated regulator of NF-kappaB pathways. Mol Cell (2004) 14:303–17. 10.1016/S1097-2765(04)00216-3 15125834

[37] Beaulieu JM Sotnikova TD Marion S Lefkowitz RJ Gainetdinov RR Caron MG. An Akt/beta-arrestin 2/PP2A signaling complex mediates dopaminergic neurotransmission and behavior. Cell (2005) 122:261–73. 10.1016/j.cell.2005.05.012 16051150

[38] Shenoy SK Drake MT Nelson CD Houtz DA Xiao K Madabushi S. beta-arrestin-dependent, G protein-independent ERK1/2 activation by the beta2 adrenergic receptor. J Biol Chem. (2006) 281:1261–73. 10.1074/jbc.M506576200 16280323

[39] Ahn S Kim J Hara MR Ren XR Lefkowitz RJ. {beta}-arrestin-2 mediates anti-apoptotic signaling through regulation of BAD phosphorylation. J Biol Chem. (2009) 284:8855–65. 10.1074/jbc.M808463200 19171933 PMC2659243

[40] Kendall RT Lee MH Pleasant DL Robinson K Kuppuswamy D McDermott PJ. Arrestin-dependent angiotensin AT1 receptor signaling regulates Akt and mTor-mediated protein synthesis. J Biol Chem. (2014) 289:26155–66. 10.1074/jbc.M114.595728 25081544 PMC4176252

[41] Eichel K Jullie D Von Zastrow M. β-Arrestin drives MAP kinase signalling from clathrin-coated structures after GPCR dissociation. Nat Cell Biol. (2016) 18:303–10. 10.1038/ncb3307 26829388 PMC4767649

[42] Zaffran S Kelly RG Meilhac SM Buckingham ME Brown NA. Right ventricular myocardium derives from the anterior heart field. Circul Res. (2004) 95:261–8. 10.1161/01.RES.0000136815.73623.BE 15217909

[43] Walker LA Buttrick PM. The right ventricle: biologic insights and response to disease: updated. Curr Cardiol Rev. (2013) 9:73–81. 10.2174/157340313805076296 23092273 PMC3584309

[44] Roche SL Redington AN. The failing right ventricle in congenital heart disease. Can J Cardiol. (2013) 29:768–78. 10.1016/j.cjca.2013.04.018 23790549

[45] Redington AN Gray HH Hodson ME Rigby M Oldershaw P. Characterisation of the normal right ventricular pressure-volume relation by biplane angiography and simultaneous micromanometer pressure measurements. Heart (1988) 59:23–30. 10.1136/hrt.59.1.23 3342146 PMC1277068

[46] Friedberg MK Redington AN. Right versus left ventricular failure: differences, similarities, and interactions. Circulation (2014) 129:1033–44. 10.1161/CIRCULATIONAHA.113.001375 24589696

[47] Wang GY McCloskey DT Turcato S Swigart PM Simpson PC Baker AJ. Contrasting inotropic responses to alpha1-adrenergic receptor stimulation in left versus right ventricular myocardium. Am J Physiol Heart Circ Physiol (2006) 291:H2013–7. 10.1152/ajpheart.00167.2006 16731650

[48] Irlbeck M Muhling O Iwai T Zimmer HG. Different response of the rat left and right heart to norepinephrine. Cardiovasc Res. (1996) 31:157–62. 10.1016/S0008-6363(95)00188-3 8849601

[49] Reddy S Zhao M Hu DQ Fajardo G Hu S Ghosh Z. Dynamic microRNA expression during the transition from right ventricular hypertrophy to failure. Physiol Genomics (2012) 44:562–75. 10.1152/physiolgenomics.00163.2011 22454450 PMC3426410

[50] Haddad F Doyle R Murphy DJ Hunt SA. Right ventricular function in cardiovascular disease, part II: pathophysiology, clinical importance, and management of right ventricular failure. Circulation (2008) 117:1717–31. 10.1161/CIRCULATIONAHA.107.653584 18378625

[51] Haddad F Hunt SA Rosenthal DN Murphy DJ. Right ventricular function in cardiovascular disease, part I: anatomy, physiology, aging, and functional assessment of the right ventricle. Circulation (2008) 117:1436–48. 10.1161/CIRCULATIONAHA.107.653576 18347220

[52] Kuc RE Carlebur M Maguire JJ Yang P Long L Toshner M. Modulation of endothelin receptors in the failing right ventricle of the heart and vasculature of the lung in human pulmonary arterial hypertension. Life Sci. (2014) 118:391–6. 10.1016/j.lfs.2014.02.020 24582810 PMC4288792

[53] Bós DDSG Van Der Bruggen CE Kurakula K Sun X-Q Casali KR Casali AG Contribution of impaired parasympathetic activity to right ventricular dysfunction and pulmonary vascular remodeling in pulmonary arterial hypertension. Circulation (2018) 137:910–24. 10.1161/CIRCULATIONAHA.117.027451 29167228

[54] Ishikawa S Honda M Yamada S Morioka S Moriyama K. Biventricular down-regulation of beta-adrenergic receptors in right ventricular hypertrophy induced by monocrotaline. Jpn Circ J. (1991) 55:1077–85. 10.1253/jcj.55.1077 1660939

[55] Sun F Lu Z Zhang Y Geng S Xu M Xu L Stage-dependent changes of β2-adrenergic receptor signaling in right ventricular remodeling in monocrotaline-induced pulmonary arterial hypertension. Int J Mol Med. (2018) 41:2493–504. 10.3892/ijmm.2018.3449 29393391 PMC5846663

[56] Leineweber K Seyfarth T Abraham G Gerbershagen H-P Heinroth-Hoffmann I Pönicke K. Cardiac β-adrenoceptor changes in monocrotaline-treated rats: differences between membrane preparations from whole ventricles and isolated ventricular cardiomyocytes. J Cardiovasc Pharmacol. (2003) 41:333–42. 10.1097/00005344-200303000-00001 12605011

[57] Seyfarth T Gerbershagen H-P Giessler C Leineweber K Heinroth-Hoffmann I Pönicke K. The cardiac β-adrenoceptor-G-protein (s)-adenylyl cyclase system in monocrotaline-treated rats. J Mol Cell Cardiol. (2000) 32:2315–26. 10.1006/jmcc.2000.1262 11113007

[58] Hahnova K Kasparova D Zurmanova J Neckar J Kolar F Novotny J. β-Adrenergic signaling in rat heart is similarly affected by continuous and intermittent normobaric hypoxia. Gen Physiol Biophys. (2016) 35:165–73. 10.4149/gpb_2015053 26891273

[59] Chen EP Akhter SA Bittner HB Koch WJ Davis RD. Molecular and functional mechanisms of right ventricular adaptation in chronic pulmonary hypertension. Ann Thorac Surg. (1999) 67:1053–8. 10.1016/S0003-4975(99)00142-3 10320250

[60] Baandrup JD Markvardsen LH Peters CD Schou UK Jensen JL Magnusson NE. Pressure load: the main factor for altered gene expression in right ventricular hypertrophy in chronic hypoxic rats. PLoS ONE (2011) 6:e15859. 10.1371/journal.pone.0015859 21246034 PMC3016335

[61] Ueno M Miyauchi T Sakai S Kobayashi T Goto K Yamaguchi I. Effects of physiological or pathological pressure load *in vivo* on myocardial expression of ET-1 and receptors. Am J Physiol Regul Integr Compar Physiol. (1999) 277:R1321–30. 10.1152/ajpregu.1999.277.5.R1321 10564203

[62] Jasmin J-F Cernacek P Dupuis J. Activation of the right ventricular endothelin (ET) system in the monocrotaline model of pulmonary hypertension: response to chronic ETA receptor blockade. Clin Sci. (2003) 105:647–53. 10.1042/CS20030139 12823096

[63] Li H Chen S Chen Y Meng QC Durand J Oparil S. Enhanced endothelin-1 and endothelin receptor gene expression in chronic hypoxia. J Appl Physiol. (1994) 77:1451–9. 10.1152/jappl.1994.77.3.1451 7836152

[64] Nielsen EA Sun M Honjo O Hjortdal VE Redington AN Friedberg MK. Dual endothelin receptor blockade abrogates right ventricular remodeling and biventricular fibrosis in isolated elevated right ventricular afterload. PLoS ONE (2016) 11:e0146767. 10.1371/journal.pone.0146767 26765263 PMC4713098

[65] Adamy C Oliviero P Eddahibi S Rappaport L Samuel J-L Teiger E. Cardiac modulations of ANG II receptor expression in rats with hypoxic pulmonary hypertension. Am J Physiol Heart Circ Physiol. (2002) 283:H733–40. 10.1152/ajpheart.01088.2001 12124222

[66] Ahn BH Park HK Cho HG Lee HA Lee YM Yang EK. Estrogen and enalapril attenuate the development of right ventricular hypertrophy induced by monocrotaline in ovariectomized rats. J Kor Med Sci. (2003) 18:641. 10.3346/jkms.2003.18.5.641 14555814 PMC3055117

[67] Chichger H Vang A O'connell KA Zhang P Mende U Harrington EO. PKC δ and βII regulate angiotensin II-mediated fibrosis through p38: a mechanism of RV fibrosis in pulmonary hypertension. Am J Physiol Lung Cell Mol Physiol. (2015) 308:L827–36. 10.1152/ajplung.00184.2014 25659900 PMC4398873

[68] Borgdorff MA Bartelds B Dickinson MG Steendijk P Berger RM A cornerstone of heart failure treatment is not effective in experimental right ventricular failure. Int J Cardiol. (2013) 169:183–9. 10.1016/j.ijcard.2013.08.102 24067600

[69] Park HK Park SJ Kim CS Paek YW Lee JU Lee WJ. Enhanced gene expression of renin–angiotensin system, TGF-β1, endothelin-1 and nitric oxide synthase in right-ventricular hypertrophy. Pharmacol Res. (2001) 43:265–73. 10.1006/phrs.2000.0777 11401419

[70] Rouleau JL Kapuku G Pelletier S Gosselin H Adam A Gagnon C. Cardioprotective effects of ramipril and losartan in right ventricular pressure overload in the rabbit: importance of kinins and influence on angiotensin II type 1 receptor signaling pathway. Circulation (2001) 104:939–44. 10.1161/hc3401.093149 11514383

[71] Falcao-Pires I Gonçalves N Henriques-Coelho T Moreira-Gonçalves D Roncon-Albuquerque R Jr. Apelin decreases myocardial injury and improves right ventricular function in monocrotaline-induced pulmonary hypertension. Am J Physiol Heart Circ Physiol. (2009) 296:H2007–14. 10.1152/ajpheart.00089.2009 19346461

[72] Yang P Kuc RE Buonincontri G Southwood M Torella R Upton PD Elabela/toddler is an endogenous agonist of the apelin apj receptor in the adult cardiovascular system, and exogenous administration of the peptide compensates for the downregulation of its expression in pulmonary arterial hypertensionclinical perspective. Circulation (2017) 135:1160–73. 10.1161/CIRCULATIONAHA.116.023218 28137936 PMC5363837

[73] Waehre A Vistnes M Sjaastad I Nygård S Husberg C Lunde IG. Chemokines regulate small leucine-rich proteoglycans in the extracellular matrix of the pressure-overloaded right ventricle. J Appl Physiol. (2012) 112:1372–82. 10.1152/japplphysiol.01350.2011 22345433

[74] Zagorski J Sanapareddy N Gellar MA Kline JA Watts JA. Transcriptional profile of right ventricular tissue during acute pulmonary embolism in rats. Physiol Genomics (2008) 34:101–11. 10.1152/physiolgenomics.00261.2007 18430806

[75] Ishikawa K Hashimoto H Mitani S Toki Y Okumura K Ito T. Enalapril improves heart failure induced by monocrotaline without reducing pulmonary hypertension in rats: roles of preserved myocardial creatine kinase and lactate dehydrogenase isoenzymes. Int J Cardiol. (1995) 47:225–33. 10.1016/0167-5273(94)02198-R 7721499

[76] Brunner F. Cardiac endothelin and big endothelin in right-heart hypertrophy due to monocrotaline-induced pulmonary hypertension in rat. Cardiovasc Res. (1999) 44:197–206. 10.1016/S0008-6363(99)00155-8 10615403

[77] Dias-Neto M Luísa-Neves A Pinho S Gonçalves N Mendes M Eloy C. Pathophysiology of infantile pulmonary arterial hypertension induced by monocrotaline. Pediatr Cardiol. (2015) 36:1000–13. 10.1007/s00246-015-1111-y 25608696

[78] Miyauchi T Yorikane R Sakai S Sakurai T Okada M Nishikibe M. Contribution of endogenous endothelin-1 to the progression of cardiopulmonary alterations in rats with monocrotaline-induced pulmonary hypertension. Circ Res. (1993) 73:887–97. 10.1161/01.RES.73.5.887 8403258

[79] Morrell NWM Danilov S Satyan KB Morris KG Stenmark KR. Right ventricular angiotensin converting enzyme activity and expression is increased during hypoxic pulmonary hypertension. Cardiovasc Res. (1997) 34:393–403. 10.1016/S0008-6363(97)00049-7 9205554

[80] Yamane T Fujii Y Orito K Osamura K Kanai T Wakao Y. Comparison of the effects of candesartan cilexetil and enalapril maleate on right ventricular myocardial remodeling in dogs with experimentally induced pulmonary stenosis. Am J Vet Res. (2008) 69:1574–9. 10.2460/ajvr.69.12.1574 19046003

[81] Andersen CU Markvardsen LH Hilberg O Simonsen U. Pulmonary apelin levels and effects in rats with hypoxic pulmonary hypertension. Respir Med. (2009) 103:1663–71. 10.1016/j.rmed.2009.05.011 19539454

[82] Frump AL Goss KN Vayl A Albrecht M Fisher A Tursunova R. Estradiol improves right ventricular function in rats with severe angioproliferative pulmonary hypertension: effects of endogenous and exogenous sex hormones. Am J Physiol Lung Cell Mol Physiol. (2015) 308:L873–90. 10.1152/ajplung.00006.2015 25713318 PMC4421786

[83] Gomez-Arroyo J Sakagami M Syed AA Farkas L Van Tassell B Kraskauskas D. Iloprost reverses established fibrosis in experimental right ventricular failure. Eur Respir J. (2015) 45:449–62. 10.1183/09031936.00188013 25261325

[84] Zagorski J Gellar MA Obraztsova M Kline JA Watts JA. Inhibition of CINC-1 decreases right ventricular damage caused by experimental pulmonary embolism in rats. J Immunol. (2007) 179:7820–6. 10.4049/jimmunol.179.11.7820 18025228

[85] Watts JA Zagorski J Gellar MA Stevinson BG Kline JA. Cardiac inflammation contributes to right ventricular dysfunction following experimental pulmonary embolism in rats. J Mol Cell Cardiol. (2006) 41:296–307. 10.1016/j.yjmcc.2006.05.011 16814320

[86] Vikholm P Schiller P Hellgren L. A modified Glenn shunt reduces venous congestion during acute right ventricular failure due to pulmonary banding: a randomized experimental study. Inter Cardiovasc Thorac Surg. (2014) 18:418–25. 10.1093/icvts/ivt547 24396048 PMC3957294

[87] Morrell NW Morris KG Stenmark KR. Role of angiotensin-converting enzyme and angiotensin II in development of hypoxic pulmonary hypertension. Am J Physiol. (1995) 269:H1186–94. 7485548 10.1152/ajpheart.1995.269.4.H1186

[88] Okada M Kikuzuki R Harada T Hori Y Yamawaki H Hara Y. Captopril attenuates matrix metalloproteinase-2 and-9 in monocrotaline-induced right ventricular hypertrophy in rats. J Pharmacol Sci. (2008) 108:487–94. 10.1254/jphs.08174FP 19057128

[89] Kanno S Wu Y-JL Lee PC Billiar TR Ho C. Angiotensin-converting enzyme inhibitor preserves p21 and endothelial nitric oxide synthase expression in monocrotaline-induced pulmonary arterial hypertension in rats. Circulation (2001) 104:945–50. 10.1161/hc3401.093155 11514384

[90] Andersen S Schultz JG Andersen A Ringgaard S Nielsen JM Holmboe S. Effects of bisoprolol and losartan treatment in the hypertrophic and failing right heart. J Cardiac Fail. (2014) 20:864–73. 10.1016/j.cardfail.2014.08.003 25135110

[91] De Man FS Tu L Handoko ML Rain S Ruiter G François C. Dysregulated renin–angiotensin–aldosterone system contributes to pulmonary arterial hypertension. Am J Respir Crit Care Med. (2012) 186:780–9. 10.1164/rccm.201203-0411OC 22859525 PMC5104838

[92] Okada M Harada T Kikuzuki R Yamawaki H Hara Y. Effects of telmisartan on right ventricular remodeling induced by monocrotaline in rats. J Pharmacol Sci. (2009) 111:193–200. 10.1254/jphs.09112FP 19809219

[93] Wu B You T Zhao H Guo Q Lian Y Ouyang Q Effects of valsartan on monocrotaline-induced right ventricular-pulmonary arterial uncoupling. Int J Clin Exp Med. (2018) 11:603–11.

[94] Bruce E Shenoy V Rathinasabapathy A Espejo A Horowitz A Oswalt A. Selective activation of angiotensin AT2 receptors attenuates progression of pulmonary hypertension and inhibits cardiopulmonary fibrosis. Br J Pharmacol. (2015) 172:2219–31. 10.1111/bph.13044 25522140 PMC4403089

[95] Underwood D Bochnowicz S Osborn R Luttmann M Louden C Hart T. Effect of SB 217242 on hypoxia-induced cardiopulmonary changes in the high altitude-sensitive rat. Pulm Pharmacol Therap. (1999) 12:13–26. 10.1006/pupt.1999.0158 10208832

[96] Nishida M Eshiro K Okada Y Takaoka M Matsumura Y. Roles of endothelin ETA and ETB receptors in the pathogenesis of monocrotaline-induced pulmonary hypertension. J Cardiovasc Pharmacol. (2004) 44:187–91. 10.1097/00005344-200408000-00007 15243299

[97] Schroll S Arzt M Sebah D Stoelcker B Luchner A Budweiser S. Effects of selective and unselective endothelin-receptor antagonists on prostacyclin synthase gene expression in experimental pulmonary hypertension. Scand J Clin Lab Invest. (2008) 68:270–6. 10.1080/00365510701673375 18612919

[98] Jasmin J-F Lucas M Cernacek P Dupuis J Effectiveness of a nonselective ETA/B and a selective ETA antagonist in rats with monocrotaline-induced pulmonary hypertension. Circulation (2001) 103:314–8. 10.1161/01.CIR.103.2.314 11208695

[99] Kim K-H Kim H-K Chan SY Kim Y-J Sohn D-W. Hemodynamic and histopathologic benefits of early treatment with macitentan in a rat model of pulmonary arterial hypertension. Korean Circ J. (2016) 48:839–853. 10.4070/kcj.2017.0394 30088353 PMC6110709

[100] Temple I Monfredi O Quigley G Schneider H Zi M Cartwright E. Macitentan treatment retards the progression of established pulmonary arterial hypertension in an animal model. Int J Cardiol. (2014) 177:423–8. 10.1016/j.ijcard.2014.09.005 25305681 PMC4251701

[101] Choudhary G Troncales F Martin D Harrington EO Klinger JR. Bosentan attenuates right ventricular hypertrophy and fibrosis in normobaric hypoxia model of pulmonary hypertension. J Heart Lung Transpl. (2011) 30:827–33. 10.1016/j.healun.2011.03.010 21550822 PMC3536478

[102] Ueno M Miyauchi T Sakai S Goto K Yamaguchi I. Endothelin-A-receptor antagonist and oral prostacyclin analog are comparably effective in ameliorating pulmonary hypertension and right ventricular hypertrophy in rats. J Cardiovasc Pharmacol. (2000) 36:S305–10. 10.1097/00005344-200036051-00089 11078405

[103] Mouchaers KT Schalij I Versteilen AM Hadi AM Van Nieuw Amerongen GP Van Hinsbergh VW. Endothelin receptor blockade combined with phosphodiesterase-5 inhibition increases right ventricular mitochondrial capacity in pulmonary arterial hypertension. Am J Physiol Heart Circul Physiol. (2009) 297:H200–7. 10.1152/ajpheart.00893.2008 19395550

[104] Brunner F Wölkart G Haleen S. Defective intracellular calcium handling in monocrotaline-induced right ventricular hypertrophy: protective effect of long-term endothelin-A receptor blockade with 2-benzo [1, 3] dioxol-5-yl-3-benzyl-4-(4-methoxy-phenyl-)-4-oxobut-2-enoate-sodium (PD 155080). J Pharmacol Exp Therap. (2002) 300:442–9. 10.1124/jpet.300.2.442 11805203

[105] Miyauchi T Sato R Sakai S Kobayashi T Ueno M Kondo H. Endothelin-1 and right-sided heart failure in rats: effects of an endothelin receptor antagonist on the failing right ventricle. J Cardiovasc Pharmacol. (2000) 36:S327–30. 10.1097/00005344-200036051-00095 11078411

[106] Drozd K Ahmadi A Deng Y Jiang B Petryk J Thorn S. Effects of an endothelin receptor antagonist, Macitentan, on right ventricular substrate utilization and function in a Sugen 5416/hypoxia rat model of severe pulmonary arterial hypertension. J Nuclear Cardiol. (2017) 24:1979–89. 10.1007/s12350-016-0663-4 27688036

[107] Ramos SR Pieles G Sun M Slorach C Hui W Friedberg MK. Early versus late cardiac remodeling during right ventricular pressure load and impact of preventive versus rescue therapy with endothelin-1 receptor blockers. J Appl Physiol. (2018) 124:1349–62. 10.1152/japplphysiol.00975.2017 29446710

[108] Iglarz M Landskroner K Bauer Y Vercauteren M Rey M Renault B. Comparison of macitentan and bosentan on right ventricular remodeling in a rat model of non-vasoreactive pulmonary hypertension. J Cardiovasc Pharmacol. (2015) 66:457. 10.1097/FJC.0000000000000296 26230396 PMC4632117

[109] De Man FS Handoko ML Van Ballegoij JJ Schalij I Bogaards SJ Postmus PE. Bisoprolol delays progression towards right heart failure in experimental pulmonary hypertension. Circ Heart Fail. (2012) 5:97–105. 10.1161/CIRCHEARTFAILURE.111.964494 22157723

[110] Fowler ED Drinkhill MJ Stones R White E. Diastolic dysfunction in pulmonary artery hypertension: creatine kinase and the potential therapeutic benefit of beta-blockers. Clin Exp Pharmacol Physiol (2018) 45:384–9. 10.1111/1440-1681.12898 29193283 PMC5887930

[111] Drake JI Gomez-Arroyo J Dumur CI Kraskauskas D Natarajan R Bogaard HJ. Chronic carvedilol treatment partially reverses the right ventricular failure transcriptional profile in experimental pulmonary hypertension. Physiol Genomics (2013) 45:449–61. 10.1152/physiolgenomics.00166.2012 23632417 PMC3680777

[112] Ishikawa M Sato N Asai K Takano T Mizuno K. Effects of a pure alpha/beta-adrenergic receptor blocker on monocrotaline-induced pulmonary arterial hypertension with right ventricular hypertrophy in rats. Circ J. (2009) 73:2337–41. 10.1253/circj.CJ-09-0213 19822980

[113] Perros F Ranchoux B Izikki M Bentebbal S Happé C Antigny F. Nebivolol for improving endothelial dysfunction, pulmonary vascular remodeling, and right heart function in pulmonary hypertension. J Am Coll Cardiol. (2015) 65:668–80. 10.1016/j.jacc.2014.11.050 25677428

[114] Hironaka E Hongo M Sakai A Mawatari E Terasawa F Okumura N. Serotonin receptor antagonist inhibits monocrotaline-induced pulmonary hypertension and prolongs survival in rats. Cardiovasc Res. (2003) 60:692–9. 10.1016/j.cardiores.2003.09.023 14659815

[115] Zhang E Maruyama J Yokochi A Mitani Y Sawada H Nishikawa M. Sarpogrelate hydrochloride, a serotonin 5HT2A receptor antagonist, ameliorates the development of chronic hypoxic pulmonary hypertension in rats. J Anesth. (2015) 29:715–23. 10.1007/s00540-015-2015-y 25931318

[116] Zopf DA Das Neves LA Nikula KJ Huang J Senese PB Gralinski MR. C-122, a novel antagonist of serotonin receptor 5-HT2B, prevents monocrotaline-induced pulmonary arterial hypertension in rats. Eur J Pharmacol. (2011) 670:195–203. 10.1016/j.ejphar.2011.08.015 21914448

[117] Janssen W Schymura Y Novoyatleva T Kojonazarov B Boehm M Wietelmann A. 5-HT2B receptor antagonists inhibit fibrosis and protect from RV heart failure. BioMed Res Int. (2015) 2015. 10.1155/2015/438403 25667920 PMC4312574

[118] Geng J Fan FL He S Liu Y Meng Y Tian H. The effects of the 5-HT2A receptor antagonist sarpogrelate hydrochloride on chronic hypoxic pulmonary hypertension in rats. Exp Lung Res. (2016) 42:190–8. 10.1080/01902148.2016.1181122 27191897

[119] Keegan A Morecroft I Smillie D Hicks MN MacLean MR Contribution of the 5-HT1B receptor to hypoxia-induced pulmonary hypertension: converging evidence using 5-HT1B-receptor knockout mice and the 5-HT1B/1D-receptor antagonist GR127935. Circ Res. (2001) 89:1231–9. 10.1161/hh2401.100426 11739290

[120] Marcos E Adnot S Pham MH Nosjean A Raffestin B Hamon M. Serotonin transporter inhibitors protect against hypoxic pulmonary hypertension. Am J Respir Crit Care Med. (2003) 168:487–93. 10.1164/rccm.200210-1212OC 12773327

[121] Chaudhary KR Deng Y Suen CM Taha M Petersen TH Mei SH Efficacy of treprostinil in the SU5416-hypoxia model of severe pulmonary arterial hypertension: hemodynamic benefits are not associated with improvements in arterial remodelling. Br J Pharmacol. (2018) 175:3976–89. 10.1111/bph.14472 30098019 PMC6151330

[122] Nikam VS Schermuly RT Dumitrascu R Weissmann N Kwapiszewska G Morrell N. Treprostinil inhibits the recruitment of bone marrow derived circulating fibrocytes in chronic hypoxic pulmonary hypertension. Eur Respir J. (2010) 36:1302–14. 10.1183/09031936.00028009 20525716

[123] Schermuly RT Yilmaz HS Ghofrani HA Woyda K Pullamsetti S Schulz A. Inhaled iloprost reverses vascular remodeling in chronic experimental pulmonary hypertension. Am J Respir Crit Care Med. (2005) 172:358–63. 10.1164/rccm.200502-296OC 15879421

[124] Axelgaard S Holmboe S Ringgaard S Hillgaard TK Andersen S Hansen MS. Effects of chronic treprostinil treatment on experimental right heart hypertrophy and failure. Cardiol Young (2017) 27:90–100. 10.1017/S1047951116000160 27087410

[125] Xia J Yang L Dong L Niu M Zhang S Yang Z. Cefminox, a dual agonist of prostacyclin receptor and peroxisome proliferator-activated receptor-gamma identified by virtual screening, has therapeutic efficacy against hypoxia-induced pulmonary hypertension in rats. Front Pharmacol. (2018) 9:134. 10.3389/fphar.2018.00134 29527168 PMC5829529

[126] Chester AH Yacoub MH. The role of endothelin-1 in pulmonary arterial hypertension. Glob Cardiol Sci Pract. (2014) 2014:62–78. 10.5339/gcsp.2014.29 25405182 PMC4220438

[127] Kelland NF Webb DJ. Clinical trials of endothelin antagonists in heart failure: publication is good for the public health. Heart (2007) 93:2–4. 10.1136/hrt.2006.089250 17170334 PMC1861328

[128] Davenport AP Hyndman KA Dhaun N Southan C Kohan DE Pollock JS. Endothelin. Pharmacol Rev. (2016) 68:357–418. 10.1124/pr.115.011833 26956245 PMC4815360

[129] Pawson AJ Sharman JL Benson HE Faccenda E Alexander SP Buneman OP. The IUPHAR/BPS Guide to PHARMACOLOGY: an expert-driven knowledgebase of drug targets and their ligands. Nucleic Acids Res. (2014) 42:D1098–106. 10.1093/nar/gkt1143 24234439 PMC3965070

[130] Asakura M Kitakaze M Takashima S Liao Y Ishikura F Yoshinaka T. Cardiac hypertrophy is inhibited by antagonism of ADAM12 processing of HB-EGF: metalloproteinase inhibitors as a new therapy. Nat Med. (2002) 8:35–40. 10.1038/nm0102-35 11786904

[131] Sugden PH. An overview of endothelin signaling in the cardiac myocyte. J Mol Cell Cardiol. (2003) 35:871–86. 10.1016/S0022-2828(03)00153-6 12878473

[132] Kawamura T Ono K Morimoto T Akao M Iwai-Kanai E Wada H. Endothelin-1-dependent nuclear factor of activated T lymphocyte signaling associates with transcriptional coactivator p300 in the activation of the B cell leukemia-2 promoter in cardiac myocytes. Circ Res. (2004) 94:1492–9. 10.1161/01.RES.0000129701.14494.52 15117818

[133] Stewart DJ Levy RD Cernacek P Langleben D Increased plasma endothelin-1 in pulmonary hypertension: marker or mediator of disease? Ann Intern Med. (1991) 114:464–9. 10.7326/0003-4819-114-6-464 1994793

[134] Kumar P Kazzi NJ Shankaran S. Plasma immunoreactive endothelin-1 concentrations in infants with persistent pulmonary hypertension of the newborn. Am J Perinatol. (1996) 13:335–41. 10.1055/s-2007-994352 8865978

[135] Nootens M Kaufmann E Rector T Toher C Judd D Francis GS. Neurohormonal activation in patients with right ventricular failure from pulmonary hypertension: relation to hemodynamic variables and endothelin levels. J Am Coll Cardiol. (1995) 26:1581–5. 10.1016/0735-1097(95)00399-1 7594089

[136] Galiè N Humbert M Vachiery J-L Gibbs S Lang I Torbicki A. 2015 ESC/ERS guidelines for the diagnosis and treatment of pulmonary hypertension: the joint task force for the diagnosis and treatment of pulmonary hypertension of the European Society of Cardiology (ESC) and the European Respiratory Society (ERS): endorsed by: Association for European Paediatric and Congenital Cardiology (AEPC), International Society for Heart and Lung Transplantation (ISHLT). Eur Heart J. (2015) 37:67–119. 10.1093/eurheartj/ehv317 26320113

[137] Iglarz M Binkert C Morrison K Fischli W Gatfield J Treiber A. Pharmacology of macitentan, an orally active tissue-targeting dual endothelin receptor antagonist. J Pharmacol Exp Ther. (2008) 327:736–45. 10.1124/jpet.108.142976 18780830

[138] Holscher M Schafer K Krull S Farhat K Hesse A Silter M. Unfavourable consequences of chronic cardiac HIF-1alpha stabilization. Cardiovasc Res. (2012) 94:77–86. 10.1093/cvr/cvs014 22258630

[139] Wölkart G Strömer H Brunner F. Calcium handling and role of endothelin-1 in monocrotaline right ventricular hypertrophy of the rat. J Mol Cell Cardiol. (2000) 32:1995–2005. 10.1006/jmcc.2000.1231 11040104

[140] Izumi M Miyamoto S Hori M Ozaki H Karaki H. Negative inotropic effect of endothelin-1 in the mouse right ventricle. Eur J Pharmacol. (2000) 396:109–17. 10.1016/S0014-2999(00)00218-1 10822063

[141] Nagasaka T Izumi M Hori M Ozaki H Karaki H. Positive inotropic effect of endothelin-1 in the neonatal mouse right ventricle. Eur J Pharmacol. (2003) 472:197–204. 10.1016/S0014-2999(03)01936-8 12871754

[142] Madamanchi A. β-Adrenergic receptor signaling in cardiac function and heart failure. McGill J Med. (2007) 10:99. 18523538 PMC2323471

[143] Jensen BC O'connell TD Simpson PC. (2014). Alpha-1-adrenergic receptors in heart failure: the adaptive arm of the cardiac response to chronic catecholamine stimulation. J Cardiovasc Pharmacol. 63:291. 10.1097/FJC.0000000000000032 24145181 PMC3980029

[144] Piao L Fang YH Parikh KS Ryan JJ D'souza KM Theccanat T. GRK2-mediated inhibition of adrenergic and dopaminergic signaling in right ventricular hypertrophy: therapeutic implications in pulmonary hypertension. Circulation (2012) 126:2859–69. 10.1161/CIRCULATIONAHA.112.109868 23124027 PMC4459732

[145] Fauchier L Melin A Eder V Antier D Bonnet P. Heart rate variability in rats with chronic hypoxic pulmonary hypertension. Ann Cardiol Angeiol (Paris) 55, 249–254. 10.1016/j.ancard.2006.01.005 17078260

[146] Vaillancourt M Chia P Sarji S Nguyen J Hoftman N Ruffenach G. Autonomic nervous system involvement in pulmonary arterial hypertension. Respir Res. (2017) 18:201. 10.1186/s12931-017-0679-6 29202826 PMC5715548

[147] Honda M Yamada S Goto Y Ishikawa S Yoshikane H Ishinaga Y. Biochemical and structural remodeling of collagen in the right ventricular hypertrophy induced by monocrotaline. Jpn Circ J. (1992) 56:392–403. 10.1253/jcj.56.392 1533690

[148] Bristow MR. Treatment of chronic heart failure with beta-adrenergic receptor antagonists: a convergence of receptor pharmacology and clinical cardiology. Circ Res. (2011) 109:1176–94. 10.1161/CIRCRESAHA.111.245092 22034480

[149] Okumura K Kato H Honjo O Breitling S Kuebler WM Sun M. Carvedilol improves biventricular fibrosis and function in experimental pulmonary hypertension. J Mol Med. (2015) 93:663–74. 10.1007/s00109-015-1251-9 25595602

[150] Bogaard HJ Natarajan R Mizuno S Abbate A Chang PJ Chau VQ. Adrenergic receptor blockade reverses right heart remodeling and dysfunction in pulmonary hypertensive rats. Am J Respir Crit Care Med. (2010) 182:652–60. 10.1164/rccm.201003-0335OC 20508210

[151] Fowler ED Drinkhill MJ Norman R Pervolaraki E Stones R Steer E Beta1-adrenoceptor antagonist, metoprolol attenuates cardiac myocyte Ca 2+ handling dysfunction in rats with pulmonary artery hypertension. J Mol Cell Cardiol. (2018) 120:74–83. 10.1016/j.yjmcc.2018.05.015 29807024 PMC6013283

[152] Wang G-Y Yeh C-C Jensen BC Mann MJ Simpson PC Baker AJ. Heart failure switches the RV α1-adrenergic inotropic response from negative to positive. Am J Physiol Heart Circ Physiol. (2010) 298:H913–20. 10.1152/ajpheart.00259.2009 20035030 PMC2838546

[153] Cowley PM Wang G Chang AN Makwana O Swigart PM Lovett DH. The α1A-adrenergic receptor subtype mediates increased contraction of failing right ventricular myocardium. Am J Physiol Heart Circ Physiol. (2015) 309:H888–96. 10.1152/ajpheart.00042.2015 26116709 PMC4591405

[154] Cowley PM Wang G Joshi S Swigart PM Lovett DH Simpson PC. α1A-Subtype adrenergic agonist therapy for the failing right ventricle. Am J Physiol Heart Circ Physiol. (2017) 313:H1109–18. 10.1152/ajpheart.00153.2017 28822963 PMC5814653

[155] Rothman RB Baumann MH Savage JE Rauser L Mcbride A Hufeisen SJ. Evidence for possible involvement of 5-HT(2B) receptors in the cardiac valvulopathy associated with fenfluramine and other serotonergic medications. Circulation (2000) 102:2836–41. 10.1161/01.CIR.102.23.2836 11104741

[156] MacLean MMR. The serotonin hypothesis in pulmonary hypertension revisited: targets for novel therapies (2017 Grover Conference Series). Pulm Circ. (2018) 8:2045894018759125. 10.1177/2045894018759125 29468941 PMC5826007

[157] MacLean MR Dempsie Y. The serotonin hypothesis of pulmonary hypertension revisited. Adv Exp Med Biol. (2010) 661:309–22. 10.1007/978-1-60761-500-2_20 20204739

[158] Herve P Launay JM Scrobohaci ML Brenot F Simonneau G Petitpretz P. Increased plasma serotonin in primary pulmonary hypertension. Am J Med. (1995) 99:249–54. 10.1016/S0002-9343(99)80156-9 7653484

[159] Kereveur A Callebert J Humbert M Herve P Simonneau G Launay JM. High plasma serotonin levels in primary pulmonary hypertension. Effect of long-term epoprostenol (prostacyclin) therapy. Arterioscler Thromb Vasc Biol. (2000) 20:2233–9. 10.1161/01.ATV.20.10.2233 11031209

[160] Jaffré F Bonnin P Callebert J Debbabi H Setola V Doly S. Serotonin and angiotensin receptors in cardiac fibroblasts coregulate adrenergic-dependent cardiac hypertrophy. Circ Res. (2009) 104:113–23. 10.1161/CIRCRESAHA.108.180976 19023134

[161] Mitchell JA Ahmetaj-Shala B Kirkby NS Wright WR Mackenzie LS Reed DM. Role of prostacyclin in pulmonary hypertension. Glob Cardiol Sci Pract. (2014) 2014:382–93. 10.5339/gcsp.2014.53 25780793 PMC4355513

[162] Mubarak KK. A review of prostaglandin analogs in the management of patients with pulmonary arterial hypertension. Respir Med. (2010) 104:9–21. 10.1016/j.rmed.2009.07.015 19683911

[163] Mechiche H Grassin-Delyle S Robinet A Nazeyrollas P Devillier P. Prostanoid receptors involved in regulation of the beating rate of neonatal rat cardiomyocytes. PLoS ONE (2012) 7:e45273. 10.1371/journal.pone.0045273 22984630 PMC3440323

[164] Chow KB Jones RL Wise H. Protein kinase A-dependent coupling of mouse prostacyclin receptors to Gi is cell-type dependent. Eur J Pharmacol. (2003) 474:7–13. 10.1016/S0014-2999(03)02006-5 12909190

[165] Wise H. Multiple signalling options for prostacyclin. Acta Pharmacol Sin. (2003) 24:625–30. 12852825

[166] Abramovitz M Adam M Boie Y Carriere M Denis D Godbout C. The utilization of recombinant prostanoid receptors to determine the affinities and selectivities of prostaglandins and related analogs. Biochim Biophys Acta (2000) 1483:285–93. 10.1016/S1388-1981(99)00164-X 10634944

[167] Bhattacharya M Peri KG Almazan G Ribeiro-Da-Silva A Shichi H Durocher Y. Nuclear localization of prostaglandin E2 receptors. Proc Natl Acad Sci USA. (1998) 95:15792–7. 10.1073/pnas.95.26.15792 9861049 PMC28123

[168] Bhattacharya M Peri K Ribeiro-Da-Silva A Almazan G Shichi H Hou X. Localization of functional prostaglandin E2 receptors EP3 and EP4 in the nuclear envelope. J Biol Chem. (1999) 274:15719–24. 10.1074/jbc.274.22.15719 10336471

[169] Gupta RA Tan J Krause WF Geraci MW Willson TM Dey SK. Prostacyclin-mediated activation of peroxisome proliferator-activated receptor delta in colorectal cancer. Proc Natl Acad Sci USA. (2000) 97:13275–80. 10.1073/pnas.97.24.13275 11087869 PMC27215

[170] Hatae T Wada M Yokoyama C Shimonishi M Tanabe T. Prostacyclin-dependent apoptosis mediated by PPAR delta. J Biol Chem. (2001) 276:46260–7. 10.1074/jbc.M107180200 11551955

[171] Li Y Connolly M Nagaraj C Tang B Balint Z Popper H. Peroxisome proliferator-activated receptor-beta/delta, the acute signaling factor in prostacyclin-induced pulmonary vasodilation. Am J Respir Cell Mol Biol. (2012) 46:372–9. 10.1165/rcmb.2010-0428OC 22021335

[172] Lai Y-J Pullamsetti SS Dony E Weissmann N Butrous G Banat G-A. Role of the prostanoid EP4 receptor in iloprost-mediated vasodilatation in pulmonary hypertension. Am J Respir Crit Care Med. (2008) 178:188–96. 10.1164/rccm.200710-1519OC 18467507

[173] Van Albada ME Berger RM Niggebrugge M Van Veghel R Cromme-Dijkhuis AH Schoemaker RG. Prostacyclin therapy increases right ventricular capillarisation in a model for flow-associated pulmonary hypertension. Eur J Pharmacol. (2006) 549:107–16. 10.1016/j.ejphar.2006.08.016 16978602

[174] Ueno Y Okazaki S Isogaya M Nishio S Tanaka H Kato Y. Positive inotropic and chronotropic effects of beraprost sodium, a stable analogue of prostacyclin, in isolated guinea pig myocardium. Gen Pharmacol. (1996) 27:101–3. 10.1016/0306-3623(95)00095-X 8742503

[175] Hall JE Guyton AC Mizelle HL. Role of the renin-angiotensin system in control of sodium excretion and arterial pressure. Acta Physiol Scand Suppl. (1990) 591:48–62. 2220409

[176] Wolf G. Role of reactive oxygen species in angiotensin II-mediated renal growth, differentiation, and apoptosis. Antioxid Redox Signal. (2005) 7:1337–45. 10.1089/ars.2005.7.1337 16115039

[177] Patten DA Lafleur VN Robitaille GA Chan DA Giaccia AJ Richard DE. Hypoxia-inducible factor-1 activation in nonhypoxic conditions: the essential role of mitochondrial-derived reactive oxygen species. Mol Biol Cell (2010) 21:3247–57. 10.1091/mbc.e10-01-0025 20660157 PMC2938389

[178] Mehta PK Griendling KK. Angiotensin II cell signaling: physiological and pathological effects in the cardiovascular system. Am J Physiol Cell Physiol. (2007) 292:C82–97. 10.1152/ajpcell.00287.2006 16870827

[179] Morrell NW Atochina EN Morris KG Danilov SM Stenmark KR. Angiotensin converting enzyme expression is increased in small pulmonary arteries of rats with hypoxia-induced pulmonary hypertension. J Clin Invest. (1995) 96:1823–33. 10.1172/JCI118228 7560074 PMC185819

[180] Maron BA Leopold JA. The role of the renin-angiotensin-aldosterone system in the pathobiology of pulmonary arterial hypertension (2013 Grover Conference series). Pulm Circ. (2014) 4:200–10. 10.1086/675984 25006439 PMC4070776

[181] Friedberg MK Cho MY Li J Assad RS Sun M Rohailla S. Adverse biventricular remodeling in isolated right ventricular hypertension is mediated by increased transforming growth factor-beta1 signaling and is abrogated by angiotensin receptor blockade. Am J Respir Cell Mol Biol. (2013) 49:1019–28. 10.1165/rcmb.2013-0149OC 23841477

[182] Mazzolai L Pedrazzini T Nicoud F Gabbiani G Brunner H-R Nussberger JR. Increased cardiac angiotensin II levels induce right and left ventricular hypertrophy in normotensive mice. Hypertension (2000) 35:985–91. 10.1161/01.HYP.35.4.985 10775573

[183] Bos DDSG Happé C Schalij I Pijacka W Paton JF Guignabert C Renal denervation reduces pulmonary vascular remodeling and right ventricular diastolic stiffness in experimental pulmonary hypertension. JACC Basic Transl Sci. (2017) 2:22–35. 10.1016/j.jacbts.2016.09.007 29034356 PMC5628179

[184] Boehm M Arnold N Braithwaite A Pickworth J Lu C Novoyatleva T. Eplerenone attenuates pathological pulmonary vascular rather than right ventricular remodeling in pulmonary arterial hypertension. BMC Pulm Med. (2018) 18:41. 10.1186/s12890-018-0604-x 29499691 PMC5833097

[185] Zhang R Wu Y Zhao M Liu C Zhou L Shen S. Role of HIF-1alpha in the regulation ACE and ACE2 expression in hypoxic human pulmonary artery smooth muscle cells. Am J Physiol Lung Cell Mol Physiol. (2009) 297:L631–40. 10.1152/ajplung.90415.2008 19592460

[186] Shenoy V Qi Y Katovich MJ Raizada MK. ACE2, a promising therapeutic target for pulmonary hypertension. Curr Opin Pharmacol. (2011) 11:150–5. 10.1016/j.coph.2010.12.002 21215698 PMC3075309

[187] Zisman LS Keller RS Weaver B Lin Q Speth R Bristow MR. Increased angiotensin-(1-7)-forming activity in failing human heart ventricles: evidence for upregulation of the angiotensin-converting enzyme Homologue ACE2. Circulation (2003) 108:1707–12. 10.1161/01.CIR.0000094734.67990.99 14504186

[188] Takahashi Y Haga S Ishizaka Y Mimori A. Autoantibodies to angiotensin-converting enzyme 2 in patients with connective tissue diseases. Arthritis Res Ther. (2010) 12:R85. 10.1186/ar3012 20470389 PMC2911869

[189] Dai HL Guo Y Guang XF Xiao ZC Zhang M Yin XL. The changes of serum angiotensin-converting enzyme 2 in patients with pulmonary arterial hypertension due to congenital heart disease. Cardiology (2013) 124:208–12. 10.1159/000346884 23548773

[190] Shenoy V Ferreira AJ Qi Y Fraga-Silva RA Diez-Freire C Dooies A. The angiotensin-converting enzyme 2/angiogenesis-(1-7)/Mas axis confers cardiopulmonary protection against lung fibrosis and pulmonary hypertension. Am J Respir Crit Care Med (2010) 182:1065–72. 10.1164/rccm.200912-1840OC 20581171 PMC2970847

[191] McKinney CA Fattah C Loughrey CM Milligan G Nicklin SA. Angiotensin-(1-7) and angiotensin-(1-9): function in cardiac and vascular remodelling. Clin Sci. (2014) 126:815–27. 10.1042/CS20130436 24593683

[192] Heresi GA Aytekin M Newman J Dweik RA. CXC-chemokine ligand 10 in idiopathic pulmonary arterial hypertension: marker of improved survival. Lung (2010) 188:191–7. 10.1007/s00408-010-9232-9 20186422 PMC2886668

[193] Yang T Li ZN Chen G Gu Q Ni XH Zhao ZH. Increased levels of plasma CXC-Chemokine Ligand 10, 12 and 16 are associated with right ventricular function in patients with idiopathic pulmonary arterial hypertension. Heart Lung (2014) 43:322–7. 10.1016/j.hrtlng.2014.04.016 24856224

[194] Zabini D Heinemann A Foris V Nagaraj C Nierlich P Balint Z. Comprehensive analysis of inflammatory markers in chronic thromboembolic pulmonary hypertension patients. Eur Respir J. (2014) 44:951–62. 10.1183/09031936.00145013 25034560

[195] Olsson KM Olle S Fuge J Welte T Hoeper MM Lerch C. CXCL13 in idiopathic pulmonary arterial hypertension and chronic thromboembolic pulmonary hypertension. Respir Res. (2016) 17:21. 10.1186/s12931-016-0336-5 26927848 PMC4770535

[196] Watts JA Gellar MA Obraztsova M Kline JA Zagorski J. Role of inflammation in right ventricular damage and repair following experimental pulmonary embolism in rats. Int J Exp Pathol. (2008) 89:389–99. 10.1111/j.1365-2613.2008.00610.x 18808531 PMC2613983

[197] Pilling D Vakil V Cox N Gomer RH. TNF-α-stimulated fibroblasts secrete lumican to promote fibrocyte differentiation. Proc Natl Acad Sci USA. (2015) 112:11929–34. 10.1073/pnas.1507387112 26351669 PMC4586854

[198] Yu X-H Tang Z-B Liu L-J Qian H Tang S-L Zhang D-W. Apelin and its receptor APJ in cardiovascular diseases. Clin Chim Acta (2014) 428:1–8. 10.1016/j.cca.2013.09.001 24055369

[199] Kim J. Apelin-APJ signaling: a potential therapeutic target for pulmonary arterial hypertension. Mol Cells (2014) 37:196–201. 10.14348/molcells.2014.2308 24608803 PMC3969039

[200] Chandra SM Razavi H Kim J Agrawal R Kundu RK De Jesus Perez V. Disruption of the apelin-APJ system worsens hypoxia-induced pulmonary hypertension. Arterioscl Thromb Vasc Biol. (2011) 31:814–20. 10.1161/ATVBAHA.110.219980 21233449 PMC3113525

[201] Alastalo T-P Li M De Jesus Perez V Pham D Sawada H Wang JK. Disruption of PPARγ/β-catenin–mediated regulation of apelin impairs BMP-induced mouse and human pulmonary arterial EC survival. J Clin Invest. (2011) 121:3735–46. 10.1172/JCI43382 21821917 PMC3163943

[202] Kim J Kang Y Kojima Y Lighthouse JK Hu X Aldred MA. An endothelial apelin-FGF link mediated by miR-424 and miR-503 is disrupted in pulmonary arterial hypertension. Nat Med. (2013) 19:74. 10.1038/nm.3040 23263626 PMC3540168

[203] Brash L Barnes GD Brewis MJ Church AC Gibbs SJ Howard LS. Short-term hemodynamic effects of apelin in patients with pulmonary arterial hypertension. JACC Basic Transl Sci. (2018) 3:176–86. 10.1016/j.jacbts.2018.01.013 29876530 PMC5981010

[204] Correale M Ferraretti A Monaco I Grazioli D Di Biase M Brunetti ND. Endothelin-receptor antagonists in the management of pulmonary arterial hypertension: where do we stand? Vasc Health Risk Manage. (2018) 14:253. 10.2147/VHRM.S133921 30323613 PMC6174907

[205] Lang IM Gaine SP. Recent advances in targeting the prostacyclin pathway in pulmonary arterial hypertension. Eur Respir Rev. (2015) 24:630–41. 10.1183/16000617.0067-2015 26621977 PMC9487617

[206] Perros F De Man FS Bogaard HJ Antigny F Simonneau G Bonnet S. Use of β-blockers in pulmonary hypertension. Circ Heart Fail. (2017) 10:e003703. 10.1161/CIRCHEARTFAILURE.116.003703 28364092

[207] Mercurio V Mukherjee M Tedford RJ Zamanian RT Khair RM Sato T. Improvement in right ventricular strain with ambrisentan and tadalafil upfront therapy in scleroderma-associated pulmonary arterial hypertension. Am J Respir Crit Care Med. (2018) 197:388–91. 10.1164/rccm.201704-0789LE 28661697 PMC5803650

[208] Van De Veerdonk MC Marcus JT Westerhof N Heymans MW Bogaard H-J Vonk-Noordegraaf A. Upfront combination therapy reduces right ventricular volumes in pulmonary arterial hypertension. Eur Respir J (2017) 49:1700007. 10.1183/13993003.00007-2017 28663315

[209] Bristow MR Minobe W Rasmussen R Larrabee P Skerl L Klein J. Beta-adrenergic neuroeffector abnormalities in the failing human heart are produced by local rather than systemic mechanisms. J Clin Invest. (1992) 89:803–15. 10.1172/JCI115659 1311717 PMC442925

[210] Lowes BD Minobe W Abraham WT Rizeq MN Bohlmeyer TJ Quaife RA. Changes in gene expression in the intact human heart. Downregulation of alpha-myosin heavy chain in hypertrophied, failing ventricular myocardium. J Clin Invest. (1997) 100:2315–24. 10.1172/JCI119770 9410910 PMC508428

[211] Van Campen JS De Boer K Van De Veerdonk MC Van Der Bruggen CE Allaart CP Raijmakers PG. Bisoprolol in idiopathic pulmonary arterial hypertension: an explorative study. Eur Respir J. (2016) 48:787–96. 10.1183/13993003.00090-2016 27390285

[212] Farha S Saygin D Park MM Cheong HI Asosingh K Comhair SA. Pulmonary arterial hypertension treatment with carvedilol for heart failure: a randomized controlled trial. JCI Insight (2017) 2:95240. 10.1172/jci.insight.95240 28814664 PMC5621927

[213] Yui Y Nakajima H Kawai C Murakami T. Prostacyclin therapy in patients with congestive heart failure. Am J Cardiol. (1982) 50:320–4. 10.1016/0002-9149(82)90183-7 7048886

[214] McLaughlin VV Shillington A Rich S. Survival in primary pulmonary hypertension: the impact of epoprostenol therapy. Circulation (2002) 106:1477–82. 10.1161/01.CIR.0000029100.82385.58 12234951

[215] Sitbon O Humbert M Nunes H Parent F Garcia G Hervé P. Long-term intravenous epoprostenol infusion in primary pulmonary hypertension: prognostic factors and survival. J Am Coll Cardiol. (2002) 40:780–8. 10.1016/S0735-1097(02)02012-0 12204511

[216] Brittain EL Pugh ME Wheeler LA Robbins IM Loyd JE Newman JH Prostanoids but not oral therapies improve right ventricular function in pulmonary arterial hypertension. JACC Heart Fail. (2013) 1:300–7. 10.1016/j.jchf.2013.05.004 24015376 PMC3763862

[217] Chin KM Badesch DB Robbins IM Tapson VF Palevsky HI Kim NH. Two formulations of epoprostenol sodium in the treatment of pulmonary arterial hypertension: EPITOME-1 (epoprostenol for injection in pulmonary arterial hypertension), a phase IV, open-label, randomized study. Am Heart J. (2014) 167:218–225.e211. 10.1016/j.ahj.2013.08.008 24439983

[218] Zheng Y Yang T Chen G Hu E Gu Q Xiong C. Prostanoid therapy for pulmonary arterial hypertension: a meta-analysis of survival outcomes. Eur J Clin Pharmacol. (2014) 70:13–21. 10.1007/s00228-013-1583-8 24026627

[219] Califf RM Adams KF Mckenna WJ Gheorghiade M Uretsky BF Mcnulty SE. A randomized controlled trial of epoprostenol therapy for severe congestive heart failure: the Flolan International Randomized Survival Trial (FIRST). Am Heart J. (1997) 134:44–54. 10.1016/S0002-8703(97)70105-4 9266782

[220] Gomez-Arroyo J Sandoval J Simon MA Dominguez-Cano E Voelkel NF Bogaard HJ. Treatment for pulmonary arterial hypertension–associated right ventricular dysfunction. Ann Am Thorac Soc. (2014) 11:1101–15. 10.1513/AnnalsATS.201312-425FR 25079379

[221] Vanderpool RR Desai AA Knapp SM Simon MA Abidov A Yuan JX. How prostacyclin therapy improves right ventricular function in pulmonary arterial hypertension. Eur Respir J. (2017) 50:1700764. 10.1183/13993003.00764-2017 28838981 PMC6330704

[222] Maron BA Opotowsky AR Landzberg MJ Loscalzo J Waxman AB Leopold JA. Plasma aldosterone levels are elevated in patients with pulmonary arterial hypertension in the absence of left ventricular heart failure: a pilot study. Eur J Heart Fail. (2013) 15:277–83. 10.1093/eurjhf/hfs173 23111998 PMC3576899

[223] Maron BA Waxman AB Opotowsky AR Gillies H Blair C Aghamohammadzadeh R. Effectiveness of spironolactone plus ambrisentan for treatment of pulmonary arterial hypertension (from the [ARIES] study 1 and 2 trials). Am J Cardiol. (2013) 112:720–5. 10.1016/j.amjcard.2013.04.051 23751938 PMC3906683

[224] Safdar Z Tamez E Thakur A Entman M Basant A Frost A Effects of spironolactone in pulmonary arterial hypertension: results of spiro study. Am J Respir Crit Care Med. (2016) 193:A7380.

[225] Andersen S Andersen A Nielsen-Kudsk JE. The renin–angiotensin–aldosterone-system and right heart failure in congenital heart disease. IJC Heart Vascul. (2016) 11:59–65. 10.1016/j.ijcha.2016.03.013 28616527 PMC5441351

